# NAPping PAnts (NAPPA): An open wearable solution for monitoring Infant's sleeping rhythms, respiration and posture

**DOI:** 10.1016/j.heliyon.2024.e33295

**Published:** 2024-06-21

**Authors:** Sofie de Sena, Matias Häggman, Jukka Ranta, Oleksii Roienko, Elina Ilén, Natalia Acosta, Jonna Salama, Turkka Kirjavainen, Nathan Stevenson, Manu Airaksinen, Sampsa Vanhatalo

**Affiliations:** a BABA Center, Department of Clinical Neurophysiology, Children's Hospital, Helsinki University Hospital and University of Helsinki, Helsinki, Finland; b Department of Physiology, University of Helsinki, Helsinki, Finland; c Department of Paediatrics, Children's Hospital, Helsinki University Hospital, Helsinki, Finland; d Brain Modelling Group, QIMR Berghofer Medical Research Institute, Brisbane, Australia; e Department of Materials Science and Engineering, Universitat Politècnica de Catalunya, BarcelonaTech, Barcelona, Spain; f School of Science, Department of Mathematics and Systems Analysis, Aalto University, Espoo, Finland

**Keywords:** Wearable, Infant, Sleep, Non-invasive monitoring, Human activity recognition, Respiration

## Abstract

**Study objectives:**

To develop a non-invasive and practical wearable method for long-term tracking of infants’ sleep.

**Methods:**

An infant wearable, NAPping PAnts (NAPPA), was constructed by combining a diaper cover and a movement sensor (triaxial accelerometer and gyroscope), allowing either real-time data streaming to mobile devices or offline feature computation stored in the sensor memory. A sleep state classifier (wake, N1/REM, N2/N3) was trained and tested for NAPPA recordings (*N* = 16649 epochs of 30 s), using hypnograms from co-registered polysomnography (PSG) as a training target in 33 infants (age 2 weeks to 18 months; Mean = 4). User experience was assessed from an additional group of 16 parents.

**Results:**

Overnight NAPPA recordings were successfully performed in all infants. The sleep state classifier showed good overall accuracy (78 %; Range 74–83 %) when using a combination of five features related to movement and respiration. Sleep depth trends were generated from the classifier outputs to visualise sleep state fluctuations, which closely aligned with PSG-derived hypnograms in all infants. Consistently positive parental feedback affirmed the effectiveness of the NAPPA-design.

**Conclusions:**

NAPPA offers a practical and feasible method for out-of-hospital assessment of infants’ sleep behaviour. It can directly support large-scale quantitative studies and development of new paradigms in scientific research and infant healthcare. Moreover, NAPPA provides accurate and informative computational measures for body positions, respiration rates, and activity levels, each with their respective clinical and behavioural value.

## Statement of significance

Infants' sleep is important for early development, health, and the quality of life for the entire family. Concerns related to sleep are common and manifest in infants’ daily environments, the home. However, there is a significant challenge in assessing infants' sleep at home, both in scientific research and public healthcare. Here, we present the development of a novel wearable, NAPPA (NAPping PAnts), designed to facilitate large-scale, objective, and quantitative studies on infant sleep outside of the hospital. We introduce a fully automated algorithmic analysis for visualising sleep architecture, closely aligned with the conventional hypnogram. Additionally, NAPPA analyses support measures of sleep cycles, respiration, body activity, and sleep posture, which may directly support benchmarking in various sleep-related research.

## 1 Introduction

Sleep plays a multi-directional role in the health and well-being of infants. It impacts early development, health, and the quality of life for both the infant and the parents [1,2]. Infant sleep is affected by a vast array of inherited, genetic, acquired, and environmental conditions [3]; at the same time, infants’ sleep and its quality are considered significant for a wide range of neurodevelopmental processes, including the acquisition of developmental milestones [4,5]. For instance, improved regularity of sleep state transitions is generally believed to support neurodevelopment, physical growth [6], socio-emotional performance [7], and temperament [8]. Therefore, sleep patterns and sleep-related behaviours in early infancy exhibit a complex interplay with a host of interconnected factors that collectively evolve to shape infant sleep and other aspects of neurodevelopment. Assessing these effects poses a significant challenge to scientific research and public healthcare.

Polysomnography (PSG) constitutes the gold standard for sleep assessment. PSG combines a set of physiological signals recorded overnight or during daytime sleep to generate an accurate assessment of sleep structure and behaviour [9]. However, PSG requires a sleep laboratory setting for infants, while out-of-hospital studies would provide the most ecologically relevant environment to study infant's sleep behaviour. Data collection from home environment often relies on parental interviews and questionnaires. Both are based on subjective parental perceptions [[10], [11], [12]] rather than providing objective, first-hand information about an infant's sleep. Additional methods, such as the wrist or ankle-worn actigraphy [10,11], may provide overall assessments of rest-activity cycles in older children and adults [13,14]. Interpretation of actigraphy signals is, however, challenged by several technical or physiological confounders [15], such as frequent physiological sleep twitches [15].

In the recent literature, several new non-invasive solutions have been proposed for other types of out-of-hospital recordings of infant sleep, including physiological recordings of respiration variability, electroencephalography, body movements, heart rate, and video [[16], [17], [18], [19]]. Analysis of such recordings can be automated to support the development of sleep state classifier algorithms [14,20,21], such as those based on electroencephalography (EEG) [22,23], electrocardiography (ECG) [24,25], and respiration signals [18,24,[26], [27], [28]], or video-based assessments of the same physiological functions by using auto-videosomnography approach [19,29]. Even movement signals as simple as a bed mattress or accelerometer can provide reasonably accurate sleep state classification when using tailored computational measures of respiration, posture, and body movements [18,30,31]. While many algorithms are sufficiently accurate for clinical or clinical research applications [32], a common practical challenge in such solutions is the struggle with transparency and/or explainability of the outputs [32], especially with the algorithms based on deep learning methods [33] that may show otherwise superior performance.

Taken together, the currently available out-of-hospital methods may target recognition of wake vs sleep states with variable accuracy, but they generally fail to recognize sleep states, specific sleep behaviours, or other qualities in sleep architecture (See Table 1). The video-based assessments could potentially provide feasible accuracy [12,19,34]. However, their utility is limited by the practical challenges and privacy concerns with at-home recordings [[34], [35], [36]]. Yet, an objective and reliable identification of sleep architecture would be essential to understand sleep from an infant's perspective more than the parental perspective available from the sleep diaries.

**Table 1:** A comparison of different methodologies for studying newborn sleep out-of-hospital. For NAPPA, some of the check marks are based on the content of this article and/or our own experience with its usage.

	NAPping PAnts (NAPPA)	Actigraphy	Sleep Diary	Bed Mattress Sensor (BMS)	Camera
**Uses multiple sleep-related physiological parameters** Measures several physiological parameters related to sleep behaviour, including respiration, body movement, and body position [12,18,19,34].	X	–	–	X	X
**Robustness in different environments** The technique is robust to typical variations in procedures, technical sources of artefacts, or environmental disturbances [12,18,34].	X	X	(?)	X	–
**Support for sleep state classification** The collected measures can be utilized to assess sleep states [12,18,19,34].	X	–	–	X	(X)
**Suitability for changing environments and mobile studies** The infant can be studied anywhere, even when the sleeping environment changes over time [12,34].	X	X	X	–	–
**Ease of use by non-experts** Learning the practical use is easy for both the staff in the lab and the parents at home [12,34].	X	X	X	–	–
**Supportive user experience** The technique is well accepted by all in the multiuser experience triad: the staff, the parents, and the infant [12,34].	X	X	X	(?)	(?)
**No privacy concerns** Parents and caregivers are not concerned about the potential to compromise privacy. Additionally, the raw recording data per se is inherently non-identifying [12,18,[34], [35], [36]].	X	X	(X)	X	–

We have recently developed an infant wearable (NAPping PAnts, NAPPA) by combining an everyday diaper cover with a triaxial movement sensor on the front waistline [37] to enable safe long-term recordings of respiration, movements, and body position both in and out of the hospital. In the present study, we aimed to use NAPPA recordings for further sleep assessments by training a sleep state classifier. We studied how well the infants’ sleep architectures can be assessed using automated NAPPA-derived Sleep Depth Trend (SDT), and whether the computation of features for this purpose could be performed directly in the wearable sensor. Additionally, we conducted a limited user experience study (UX group) where 16 parents were interviewed about their personal views and user experience related to NAPPA.

## 2 Methods

**Fig. 1:**
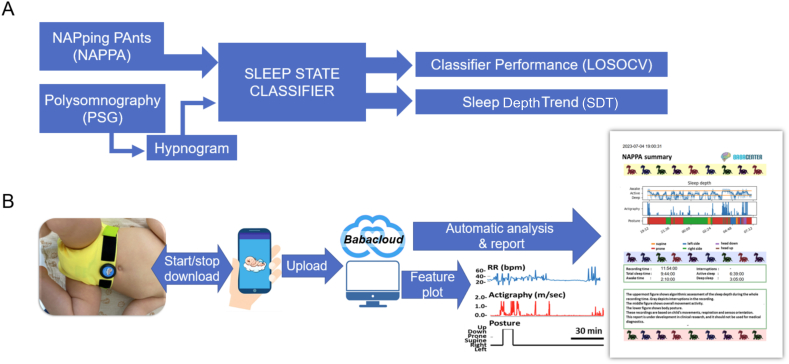
Overview of the study protocol. A) The sleep state classifier was trained for NAPPA recordings utilising hypnograms from the simultaneously recorded PSG study. The classifier performance was formally evaluated via the Leave-One-Subject-Out Cross-Validation (LOSOCV). The Sleep Depth Trend (SDT) was constructed from classifier outputs to visualise a continuous trend that depicts sleep state over time. B) The full pipeline in the NAPPA study. The NAPPA wearable with the movement sensor is shown in the picture on an infant at 12 months of age. The sensor is operated with the mobile device, which stores data until uploaded to a server. The recorded data can be directly accessed on the mobile device or uploaded for an automated analysis in the Babacloud. Automated analysis delivers results in both a graphical and numerical format for later use.

A schematic overview of the study is presented in Fig. 1 for both the classifier training (Fig. 1A) and the complete NAPPA study pipeline (Fig. 1B). The wearable recordings were performed in the sleep laboratory simultaneously with a PSG study to collate a dataset for training the sleep state classifier, thereby also authenticating the computational features required from the movement sensor. The complete study pipeline (Fig. 1B) included the NAPPA garment operated through the mobile app, followed by data transfer for further analysis, either locally or on a cloud server, incorporating the newly trained classifier algorithm. Additionally, we assessed the parental user experience (UX) in a supplementary UX group from at-home recordings.

### 2.1 Participants

The infant cohort collected for classifier training and performance testing was recruited prospectively from the infants that came to the sleep laboratory at the Department of Children's Clinical Neurophysiology, Children's Hospital, Helsinki University Hospital. These children came to the hospital for clinically indicated polysomnography (PSG) studies, and they gave permission to add NAPPA recordings to their clinical study protocol. The cohort size was N = 33 infants, with ages ranging from 2 weeks to 18 months (Median = 3 months, IQR = 1–4 months; see S7 for a detailed age distribution). The UX group (N = 16) comprised parents who had completed at least one NAPPA measure with their infant, either around 7 or 12 months of age. The measurement points exhibited some variation, ranging from 7 to 13 months, with a median age of 8 months (IQR = 7.75–10.25). Informed consent was provided by the parents in the infant cohort and UX group. The study was approved by the Ethics Committee of the New Children's Hospital, Helsinki University Central Hospital (approval number HUS/284/2019).

### 2.2 NAPPA wearable

NAPPA is a custom-made infant wearable [37] (see Fig. 1) that is designed for easy, and minimally disruptive and safe recording of sleep-relevant physiological parameters: respiration, position, and movement. The wearable consists of a textile garment shaped to cover the diaper, an embedded connector mount for a programmable movement sensor, and a detachable movement sensor. The garment can be washed in a laundry machine at 40 °C with standard detergents. The sensor is waterproof; however, it is recommended to detach it before washing the pants. For further details of the design, please refer to Ranta et al. [37].

The movement sensor (Movesense [38]; Vantaa, Finland) is a small size (diameter 36.6 mm, thickness 10.6 mm, weight 30 g) triaxial inertial measurement unit (IMU) that records multiple synchronized signals at an adjustable sampling rate. Here, we collected signals at 13Hz from the accelerometer and gyroscope (x/y/z directions), measuring linear acceleration in m/s2 (range ±8 g) and the gyroscope unit measuring angular velocity in °/s (range ±500°/s). The sensor has an open, programmable interface, wireless Bluetooth communication, a possibility for in-sensor computation, and limited data storage. It is currently available in versions certified for medical device use, as well as a technically compatible version for other health monitoring purposes; the latter was used in the present study.

### 2.3 Recordings

**NAPPA Recordings*.*** The movement data was stored either into the mobile device by real-time streaming over Bluetooth or it was logged as computational features into the sensor memory, to be downloaded to the mobile device afterward. The sampling frequency (13 Hz) and other IMU sensor specifications were similar in both modes of signal collection. The streaming mode of data collection was done over a Bluetooth connection using an open-source application programming interface (REST-based) that supports wireless transmission via Bluetooth 4.0/5.0 to an external data logging device. To this purpose, we developed an openly available, custom-built Android data-logging software (SleepSense; Android v10 or higher) [39]. For the in-sensor logging, we computed the sleep-relevant features (see Supplemental material *S1* for further details) into the sensor memory, and their logs were downloaded later using another custom-built application (NAPPA logger; Android v10 or higher). This enables phone-free recording and removes concerns related to continuous Bluetooth connections and related power consumption. All feature computation and logging in the sensor mirrored the offline feature computation performed on the raw data. In the present implementation, the feature logs can be submitted for sleep classification in the cloud (www.babacloud.fi; credentials available at request from the authors), providing the user with a graphical report as well as full analyses in CSV format (see Supplemental files S2 “Example Report.pdf” and S3 “Classifier Result.csv”). Due to memory limitations in the current sensor version, we set data logging (adjustable via the NAPPA logger application) to cover 12 h a day, typically from 7pm to 7am. This setup allows recording for at least three consecutive nights.

**PSG Recordings.** The PSG studies (Fig. 1) included continuous monitoring of four electroencephalogram-channels (Cz-Fz, Cz-O2, C4-M1, and O2-M1), two electro-oculography channels, chin and diaphragm electromyography (EMG), nasal airflow (pressure transducer), respiratory movements (thoracic and abdominal bands), electrocardiography, two pulse oximetry measurements for oxyhaemoglobin saturation (SpO2) performed in 2–4 s signal averaging (Embla or SOMNOTM HD, and Masimo Radical Pulse CO-Oximeter, Masimo Co), end-tidal carbon dioxide (EtCO2) (CAP10 Capnograph; Medlab medizinische Diagnosegeräte GmbH), transcutaneous carbon dioxide (TcCO2) (SenTec Inc.), a movement-sensor mattress (Emfit Ltd), a position sensor, and a synchronized video recordings. For PSG, the Embla N700 (Natus Medical Inc.) PSG system was used before 2018 and SOMNOTM HD (SOMNOmedics GmbH) from September 2018 onwards. We did not use a thermistor in our PSG setting.

All PSG recordings were analysed and scored by a clinical expert (T.K.) using Embla® RemLogic™ (Natus) or SOMNOmedics DOMINO software (SOMNOmedics). The expert, who was unaware of this study during the clinical sleep scoring process, peformed sleep stage and respiratory event analyses visually for every 30-s epochs according to American Academy of Sleep Medicine guidelines [40]. The recording length ranged from one to 10 h, with a median of 3.44 h (IQR = 2.73–4.98 h). Similarly, the number of epochs per recording varied from 135 to 1181, with a median of 418 (IQR = 327–599 epochs). This hypnogram was utilized for training the sleep classifier with NAPPA data.

### 2.4 Development of sleep state classifier

There were four main stages in the development of classifier and SDT outputs: 1) establishing classifier targets, 2) choosing suitable features for the present aims, 3) training and performance testing of the classifier, and 4) implementation of the SDT output. All the algorithms are freely available on GitHub and/or from the researchers at request. The classifier algorithms are freely available in Babacloud where credentials can be requested freely from the authors.

**Classifier Targets.** For classifiers intended to replace or supplement current clinical workflows, it would be logical to target conventional sleep scoring according to AASM guidelines [40], with a particular interest in REM sleep, which often reveals sleep-related breathing disorders. However, our NAPPA solution is designed to support the out-of-hospital assessment of infants’ sleep behaviour, emphasizing overall sleep structure more than discrete sleep states.

Here, we chose to focus on the distinction between three different vigilance states (Wake, N1/REM, and N2/N3) for the following reasons: First, tracking sleep state dynamics over an extended period requires the detection of states characterizing the increasing state of sleep, from wake to light sleep and further to deep sleep. Second, a reliable distinction between REM vs N1 would require measures of muscle tone and/or eye movements, while a reliable distinction between N2 vs N3 sleep would need the measurement of cortical activity by EEG [31].

We wanted to confirm this physiological reasoning [31] with our present data and assessed how well NAPPA data can support alternative sleep classifications. The results are shown in detail in Supplemental material *S4*.

**Feature Selection.** Based on our prior study [18], we chose to use five features that reflect physiological sleep behaviour and they can be reliably estimated using IMU sensor signals: Body activity was recorded from the accelerometer signals as a mean of 5-sec epochs, while the respiration-related measures were estimated from the gyroscope signals as a mean of 30-sec epochs (See Supplemental *S5* “Features.pdf” for a full description of the features). Measuring respiration from the NAPPA-integrated movement sensor relies on abdominal movements. While respiration is also present in the accelerometer signals alone [41,42], our comparative study showed that the gyroscope is more sensitive and reliable for measuring respiratory parameters from the infant's abdomen [43]. Before using it in the classifier training, the features were normalised using a combination of methods (see Supplemental material *S6* for further details).

**Fig. 2:**
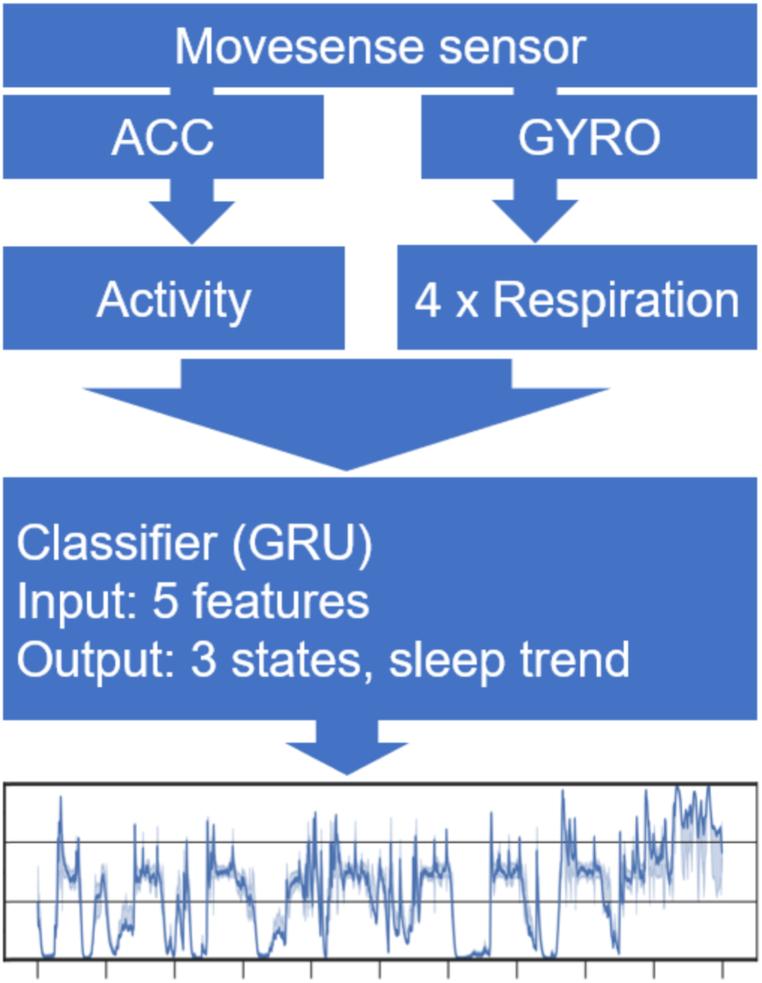
This diagram outlines the process from the Movesense sensor to sleep trend visualisation. The features are computed and stored internally within the sensor. Activity data from the accelerometer (ACC) and four distinct respiration parameters from the gyroscope (GYRO) are then fed into the classifier, generating a three-state visualisation of a sleep trend.

**Training of the Sleep State Classifier.** The full processing pipeline (shown in Fig. 2) consisted of three main blocks: feature extraction, sleep state classifier, and sleep trend visualisation. The classifier was developed in Python using the PyTorch [44] framework. Training, subsequent evaluation of the classifier performance, and classifier output visualisation were carried out on the Google Collab platform, utilising an NVIDIA T4 GPU. The classifier has been made open source and is available on GitHub [45] under the MIT licence.

We use a Bidirectional Gated Recurrent Unit (GRU) [46] with two layers, each with 10 hidden units for the classification task. The bidirectional architecture allows the model to simultaneously process data from past and future time steps, thus enhancing context. This is particularly useful in predicting time series and sequential data, as context from both previous and succeeding data points can significantly contribute to the accuracy of the predictions [47].

We also assessed the effect of two potential confounders on the classifier training: First, the infant's age is known to affect respiration rate, especially during the first two years after birth [48]. Therefore, we tested whether classifier performance could change by adding an infant's age as a feature, or by controlling for the respiration rate by subtracting age-estimated mean values (see Supplemental material *S8* for details). Neither of these made a significant difference in classifier performance. Second, classifier performance may be compromised by class imbalance, such as the under-represented “wake class” in our PSG cohort. We tested class weighting to account for class imbalance without overall improvement in the model performance, hence it was excluded from the final model. See Supplemental material *S9* for further details.

**Fig. 3:**
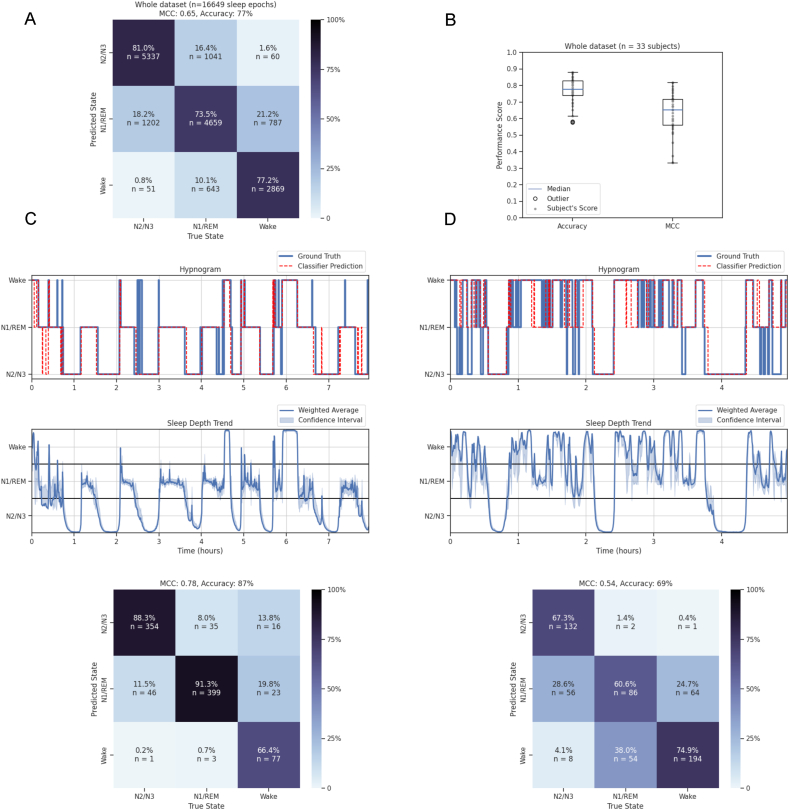
Classifier results and example Sleep Depth Trend (SDT) outputs. A) Group-level confusion matrix. B) Box plots of classifier performance with separate plots for MCC and Accuracy scores. C) An example subject with a well-developed and stable cycling in the hypnogram (top), the corresponding SDT (middle) generated from the classifier outputs, and the confusion matrix for this recording. D) An example subject with more fragmented sleep and only three clear deep sleep epochs in the hypnogram (top), the corresponding SDT (middle) generated from the classifier outputs, and the confusion matrix for this recording.

**Assessment of Classifier Performance*.*** The classifier performance was evaluated via the Leave-One-Subject-Out Cross-Validation (LOSOCV) paradigm. We calculated the accuracy score (ACC) and Matthews Correlation Coefficient (MCC) [49] both at the level of individual sleep recordings and at the group level. To provide a comprehensive and transparent view of the classifier performance, we computed confusion matrices for both the full dataset (Fig. 3A) and the individual recordings (Supplemental material *S10*). Box plots were used to illustrate the variation in classifier performance across individual sleep recordings (Fig. 3B).

**Sleep Visualisation with Sleep Depth Trend (SDT).** Classifier output was visualised for each individual by a hypnogram with discrete sleep states and as a continuous sleep depth trend (SDT); the latter depicts a weighted average of the sleep state and its confidence, showing a more smoothed and graded counterpart of the discrete-valued hypnograms (for details see Moghadam et al. [50]). Graded levels of sleep states are assumed to represent the genuine state of sleep more physiologically [51,52].

### 2.5 User experience

The UX group was requested to provide feedback through an online survey with a series of yes/no questions followed by an additional information field and two open-ended questions (Supplemental material *S11*). Further questions in the questionnaire were included to facilitate product design (not shown here).

## 3 Results

The full dataset of combined NAPPA and PSG recordings from N = 33 infants yielded a total of N = 16649 epochs with a duration of 30 s. A state-wise break-down was as follows: wake = 3716 epochs, N1 = 2220 epochs, N2 = 2316 epochs, N3 = 4274 epochs, REM = 4123 epochs, corresponding to an average of N = 505 epochs per infant (range 135–1181 epochs).

### 3.1 Classifier performance

The Leave-One-Subject-Out (LOSO) evaluation of the three-state sleep classifier across the whole cohort (33 subjects) demonstrated a median accuracy of 78 % (IQR 74–83 %) and a median Matthews Correlation Coefficient (MCC) of 0.65 (IQR 0.56–0.72). Among the sleep stages, the deep sleep (N2/N3) class achieved the highest median accuracy of 82 % (IQR 62–91 %). The light sleep (N1/REM) class had a somewhat lower accuracy at 76 % (IQR 67–86 %). The wake class showed comparable accuracy to the light sleep class (75 %; IQR 63–87 %) though the wake epochs were under-represented in this dataset with PSG studies. Little improvement in classifier performance could be seen with subject-wise normalisation (see Supplemental material (*S12*).

Inspection of the confusion matrices both at the group (Fig. 3A) and individual levels (Fig. 3CD), shows how sleep state misclassifications primarily occur between neighbouring categories. For instance, 21 % of true wake periods are classified as N1/REM, whereas 16 % of N1/REM epochs are classified as N2/N3. The full SDT time courses of individual recordings show clearly that sleep states do not change as abrupt and discrete jumps, but there is often a gradual change, especially when descending towards deeper sleep states. Our detailed visual comparison of the classifier output and the PSG-derived hypnograms suggested that most of the apparent “classification errors” occur at the state boundaries where the classifier indicates a gradual change while the clinical hypnogram (artificially) indicates stepwise changes. This is particularly prominent in this dataset that consisted of clinically indicated PSG studies that are typically characterised by prominent hypnogram fragmentation.

**Fig. 4:**
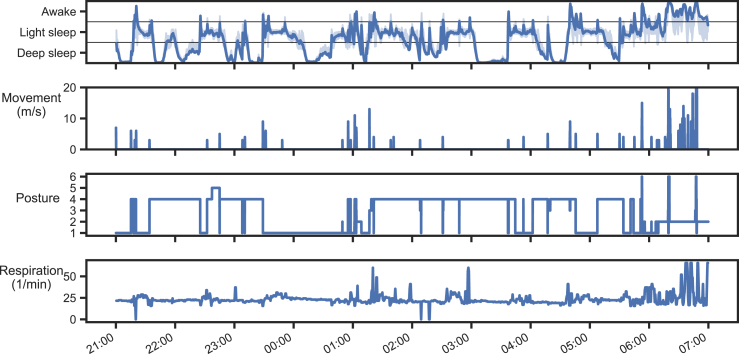
An example of the different analysis output from the automated NAPPA pipeline during a typical night recording between 9:00 p.m. to 7:00 a.m. The uppermost graph depicts Sleep Depth Trend (SDT) and its confidence (shaded) based on sleep classifier, showing somewhat irregular sleep state cycling until wake-up in the morning before 6 a.m. The second graph shows the movement activity, akin to actigraph. The third graph shows body postures, where the posture classification categories are designated as: 1 = left side, 2 = supine, 3 = right side, 4 = prone, 5 = head down, and 6 = head up. The lowest graph shows respiration rate with 30-s resolution. The sudden “spikes” in this signal are apparent artefacts due to changing posture or other movements.

Fig. 4 shows an example of all results outputs available from the NAPPA system: The sleep architecture is shown as SDT that uses classifier outputs, while the physiological parameters (movement, posture, respiration rate) can be plotted directly from the datalogs (“recordings”) that are stored in the sensor memory as a CSV matrix. In addition, the autocorrelation feature can be used as a quality control parameter to help in identifying epochs with too much confounding activity, such as changing posture or external movements.

### 3.2 User experience

All sixteen respondents (100 %) considered NAPPA safe on all occasions, reporting no observed skin marks or impact on the infant's everyday behaviour. They all also indicated willingness to use NAPPA again. One respondent (6 %) reported inadequate adhesion of the Velcro used for tightening the pants around the waist, and another respondent (6 %) noted that the textile material felt abrasive. Two respondents (13 %) mentioned specific situations when they would prefer not to use NAPPA: either when the child is unwell or during swimming. Regarding the infant's perspective as assessed by the parent, all of them (100 %) considered NAPPA comfortable for the infant, and comparable to their regular clothing. Several parents noted that the infants could show a brief curiosity about the pants at the start of the recording. Two parents (13 %) wished to have a pull-up style sleep pants with an adjustable Velcro, which was then implemented later in the project; other parents were content with the current design.

## 4 Discussion

Our present study demonstrates the potential of an easy-to-use wearable system NAPPA in assessing different aspects of infants' sleep. This method combines several previously published and utilized sleep research lines: 1) tracking of infant's movements akin to actigraphs, 2) measuring posture and respiration akin to PSG studies, and 3) classifying sleep using machine learning methods. The present findings are generally compatible with the literature in these respective areas, while NAPPA extends the prior art by showing how they can be all combined for a practical and potentially useful methodology in infant studies out-of-hospital. The key advantages of an at-home study are the significantly improved ecological validity of the study per se, and a clear the reduction of burden on the children and families.

### 4.1 Classifier performance and the sleep depth trend (SDT)

The performance of our classifier results is broadly comparable with prior research, showing good sleep state classification performance with respiration and movement signals in paediatric populations [20,53,54]. Classifying discrete sleep states in discrete duration epochs leads to an inherent ambiguity when the target (sleep) is presumably continuous in both time and state. A typical inter-rater agreement between trained human experts varies in classified scores, typically ranging from about 60 to 80 % in PSG studies with the full set of recorded signals [55]. In comparison, the reported classification accuracy from the NAPPA-based classifier is surprisingly high when considering it comes without measures of cortical, muscular, or ocular activity. This is best reconciled in the context of more recent work, including the present findings, which show that many alternative sets of physiological parameters may provide reasonably accurate sleep classifications [21,56], and the choice of signals needs to be tailored concerning specific, practical considerations in each use case, such as the casual home recordings in young infants. While some of the apparent epoch-level misclassifications are genuine classification errors, it is also essential to recognize that sleep states are not biologically discrete [51,52,57]; our classifier output, SDT, recognizes this graded nature of sleep; its actual correspondence to the real infant's sleep calls for benchmarks other than conventional, discrete sleep scores.

The present NAPPA analysis introduces a novel concept for visualising sleep architecture, the Sleep Depth Trend (SDT). It follows the idea of “hypodensity” that was recently developed in the adult sleep research, which generally matches with the conventional hypnogram taken from the manually scored discrete sleep classes by the established rules in sleep medicine [40]. However, SDT extends the conventional hypnogram approach by providing a continuous trend both in time and in sleep state, which yields a naturalistic overview of the sleep architecture over the recording period (e.g. whole night sleep). Thus, SDT directly supports a visual, easy, and intuitive assessment of the overall sleep architecture, including assessment of regularity and number of sleep cycles, or the occurrence of arousals and awakenings. It is also directly possible to extract more accurate duration metrics by thresholding the SDT. The natural, graded state of sleep is presented clearly in SDT, and it is very clearly observed in the overnight recordings when the infant transits toward deeper sleep. The SDT visualisation also depicts confidence in sleep state classification which is likely an important aspect of automated classifier outputs.

### 4.2 User experience

The ultimate uptake of wearable methods is heavily influenced by the daily user experience (UX), which in the infant context implies need to optimize a multi-user experience [58,59]: including parents, infants and the professionals (healthcare or research). Our limited UX study evaluated parental perceptions that inherently includes both parent's and infant's experience. The survey findings and a large volume of unstructured feedback (from hundreds of further studies to date; unpublished) indicate that the NAPPA design is well received, and it does not appear to interfere with infant's normal behaviour. Some technical details (e.g. Velcro attachment) were already corrected in the garment design during the present study.

### 4.3 Limitations

Our study has some limitations that mostly result from collecting the data in the sleep laboratory that is necessary for obtaining the PSG benchmark needed for the classifier training. As compared to the typical at-home recordings in the prospective NAPPA use cases, our present dataset implies two potential limitations: First, a skewed age distribution, and second, the utilisation of data collected in a PSG lab.

Our training data had proportionally more younger infants, thus the results cannot be used for generating quantified measures of age-typical sleep. The skewed age distribution is due to the fact that PSG lab studies without meaningful abnormalities (i.e. reasons to be excluded from the training dataset) are far more common during early than late infancy. It is notable, however, that we trained the sleep state classifiers for the standard epochs (30s) where the sleep classification rules do not change over the age range of interest [9]. The systematic assessments through our computational features showed that only respiration was significantly correlated with age, and its effects on the classifier performance were then excluded in particular. In contrast to the epoch-level inspection in our work, it is very well known that infants’ sleep behaviour at longer temporal scales (e.g. sleep architecture, state durations, sleep twitching etc.) is strongly affected by age, health, and other issues. Indeed, a key aim of the NAPPA method was to provide objective and openly accessible tools to study them by many other researchers in the future.

Sleep in the PSG lab typically includes less wake time and higher sleep fragmentation than what would be seen at home. The lower proportion of wake periods may have compromised classifier performance estimates for wake because classifiers typically need balanced representations of different target categories. Therefore, our model's overall reliability and accuracy may have suffered as a result of the under-representation of the waking state in our data. Future studies with well-annotated, multi-day, at-home recordings are needed for optimising wake detection. The fragmented sleep architecture in our cohort means more state transition, which challenges epoch-level classification by both human and classifier. In practice, this likely leads to underestimation of the classifier performance as compared to a typical at-home sleep with more confluent sleep states. The use of SDT as NAPPA output reduces the impact of epoch-level ambiguity, while also providing the user with an intuitive handle on assessing infants' sleep.

### 4.4 Practical aspects of future deployment

The future uptake of new health technology, such as NAPPA, depends on the combination of technical possibilities and the potential added value it may provide the users in different use cases. Regarding technical possibilities and constraints, our present solution is already used in a (clinical) research context, with hundreds of successful nights recorded in different countries. The most significant constraint is the sensor memory, which limits current recordings to about 36 h (or 12 h per day for three days) and limits the geographic distance between the lab and the infants. However, the near future introduction of large memory sensors will support many days of recordings. Recording of movement and position alone will multiply the recording time due to savings in battery and memory. The mobile application can be improved for better user experience and by developing applications for the iOS platform as needs arise.

As for scalability, the present NAPPA system is openly available (both the hardware and software), with current pricing around $100–150 per measurement unit (garment and sensor), which can be recycled at least tens of times. So, the unit price for a measurement session is only a few dollars, making staff labor the most expensive component of the study pipeline. Another significant cost is transport to/from the homes, though return can be organized using a regular envelope. For a much broader scalability to distributed geographic studies including thousands of subjects, there are some needs to optimize the hardware production and the application utility. However, those costs would still be minimal compared to the human labor in such projects.

Perhaps the most critical bottleneck in broader uptake is the need to prove the added value of the NAPPA solution in each specific use context. Such data will come from ongoing and further studies, which we hope to expedite by making the NAPPA system openly available. Most importantly, there needs to be a clear perceived added value for the user, who may be a researcher, parent, or healthcare personnel – all specific to the given use case. Moreover, novel MedTech solutions are often challenged by regulatory requirements that vary with geographic areas and the aimed use cases, with pediatric devices perceived as particularly challenging due to the relative lack of commercial interest and difficulties in conducting validation studies [60,61]. Making the NAPPA solution open to users aims to facilitate the transition from MedTech research to healthcare and clinical research [61,62].

### 4.5 Potential future research

Our work extends prior infant studies [63,64] by estimating five physiological parameters as computational features from the gyroscope and accelerometer data recorded with an IMU at the infant's waist [37]. These measures, including respiratory rate and its variability, movement activity, and sleep position, can be directly exploited in further research. The sleep profile provided by our SDT output can be used in studies where sleep disruptions are compared to neurodevelopment, sleeping environment, sleeping habits (e.g., co-sleeping or sleeping position), acute illness (e.g., infections), or maternal health [5,[65], [66], [67], [68], [69]]. Conversely, the NAPPA system may provide transparent and interpretable measures for assessing efficacy in educational and other interventions in infant sleep [70,71]. The use of SDT alone or in combination with individual measures (sleeping position, movements) will open a possibility to understanding what parental report about sleep quality (diaries) means when looking from the infant's perspective. For instance, what in an infant's sleep makes the parent perceive the sleep as “good quality” or “bad quality”? These studies are fundamental in nature, yet they have very broad practical implications as well, and they will likely depend on further computational measures, such as sleep cyclicity [72].

## Ethical Statement

The study was reviewed and approved by the Ethics Committee of the New Children's Hospital, Helsinki University Central Hospital, with the approval number: HUS/284/2019. All legal guardian provided informed consent to participate in the study, and for the publication of their anonymised case details and images. Funding Information. The work leading up to this article has received funding from the Academy of Finland (314602, 335788, 335872, 332017, 343498), the Finnish Paediatric Foundation (Lastentautiensäätiö), the Sigrid Jusélius Foundation, and the Finnish Brain Foundation (Suomen Aivosäätiöä).

## Data availability

Data can be made available on request according to legal constraints in each use case.

## CRediT authorship contribution statement

**Sofie de Sena:** Writing – review & editing, Writing – original draft, Visualization, Investigation, Formal analysis, Data curation. **Matias Häggman:** Writing – review & editing, Writing – original draft, Visualization, Validation, Software, Methodology, Investigation, Formal analysis, Data curation. **Jukka Ranta:** Software, Methodology, Investigation, Formal analysis, Data curation, Conceptualization. **Oleksii Roienko:** Writing – review & editing, Writing – original draft, Software, Methodology. **Elina Ilén:** Writing – review & editing, Methodology, Conceptualization. **Natalia Acosta:** Writing – review & editing, Methodology, Investigation. **Jonna Salama:** Writing – review & editing, Investigation. **Turkka Kirjavainen:** Writing – review & editing, Validation, Investigation, Conceptualization. **Nathan Stevenson:** Writing – review & editing, Supervision, Methodology, Investigation, Funding acquisition, Conceptualization. **Manu Airaksinen:** Writing – review & editing, Visualization, Validation, Supervision, Methodology, Investigation, Conceptualization. **Sampsa Vanhatalo:** Writing – review & editing, Writing – original draft, Visualization, Supervision, Resources, Project administration, Methodology, Investigation, Funding acquisition, Conceptualization.

## Declaration of competing interest

The authors declare the following financial interests/personal relationships which may be considered as potential competing interests:Sampsa Vanhatalo reports financial support was provided by Academy of Finland, Finland. Sampsa Vanhatalo reports financial support was provided by Sigrid Jusélius Foundation, Finland. Sampsa Vanhatalo reports financial support was provided by Finnish Pediatric Foundation, Finland. Sampsa Vanhatalo reports financial support was provided by Finnish 10.13039/501100000942Brain Foundation, Finland. If there are other authors, they declare that they have no known competing financial interests or personal relationships that could have appeared to influence the work reported in this paper.
