# Supplementary material for: NAPping PAnts (NAPPA): An open wearable solution for monitoring Infant's sleeping rhythms, respiration and posture
Source: Heliyon. 2024 Jun 21;10(13):e33295. doi: 10.1016/j.heliyon.2024.e33295 (PMC11255670; doi:10.1016/j.heliyon.2024.e33295)
Supplement: Multimedia component 1 [file mmc1.pdf]

2023-11-08 19:00:31

NAPPA summary

PILKE_110

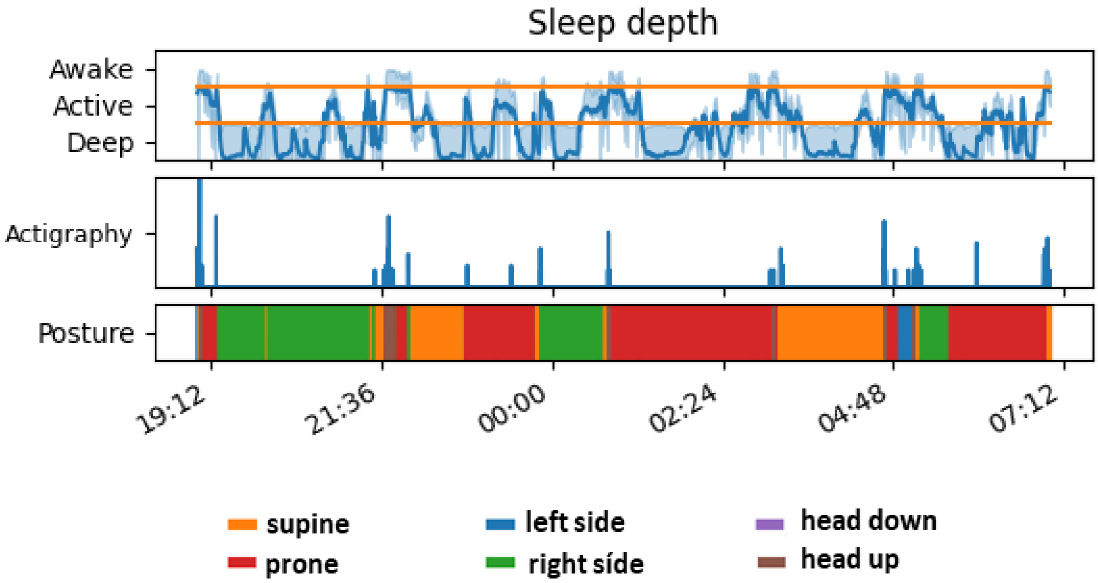

Recording time :	11:49:00	Interruptions :	-
Total sleep time :	10:01:30	Active sleep :	4:08:30
Awake time :	1:47:30	Deep sleep :	5:53:00

The uppermost figure shows algorithmic assessment of the sleep depth during the whole recording time. Gray depicts interruptions in the recording.
The middle figure shows overall movement activity.
The lower figure shows body posture.
These recordings are based on child's movements, respiration and sensos orientation.
This report is under development in clinical research, and it should not be used for medical diagnostics.
