# Supplementary material for: NAPping PAnts (NAPPA): An open wearable solution for monitoring Infant's sleeping rhythms, respiration and posture
Source: Heliyon. 2024 Jun 21;10(13):e33295. doi: 10.1016/j.heliyon.2024.e33295 (PMC11255670; doi:10.1016/j.heliyon.2024.e33295)
Supplement: Multimedia component 3 [file mmc3.pdf]

Respiration Signal Peak Characteristics

Jukka Ranta
Baba Center

September 15, 2020

Description

The peak characteristics (median and standard deviation) of the respiration signal indicate the belly movements and respiratory regularity.

Previous studies indicated that the gyroscope y-axis provides the most accurate respiratory signal for infants. The characteristics are calculated from that gyroscope channel. The channel is filtered with 0.1-1.5 Hz 2nd order Butterworth band-pass filter. Note that this preprocessing is same as in respiration autocorrelation feature calculation. From the filtered signal the peaks are found and from those peaks median value is calculated in addition to standard deviation.

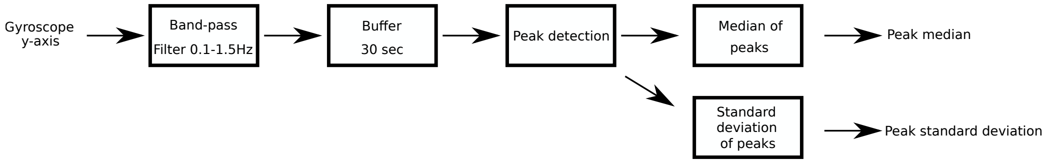

Figure 1: Flowshart of peak characteristics algorithm.

Algorithm outline

1. Band-pass filter with second order Butterworth in 0.1-1.5Hz
2. Buffer into 30 sec non-overlapping windows and for each window calculate
   (a) Find peaks using Matlab `findpeaks` function with minimum peak distance of 1 second.
   (b) Calculate meadian of the peaks
   (c) Calculate standard deviation of the peaks

Respiration Autocorrelation Calculation

Jukka Ranta
Baba Center

September 15, 2020

Description

The maximum value of autocorrelation function indicates the regularity of respiration and is a good feature to distinguish infant sleep states. From the corresponding time lag we can also derive the respiration rate.

Previous studies indicated that the gyroscope y-axis provides the most accurate respiratory signal for infants. The autocorrelation is calculated from that gyroscope channel. The channel is filtered with 0.1-1.5 Hz 2nd order Butterworth band-pass filter and the autocorrelation is calculated from 30 sec window. In Matlab source code `autocorr` function is used. The autocorrelation function is pre-processed so that all all the values before the first negative autocorrelation value is set to zero in addition to all negative values. After that the maximum peak (Matlab `findpeaks` and `max`) is taken and corresponding lag is used to calculate the respiration rate.

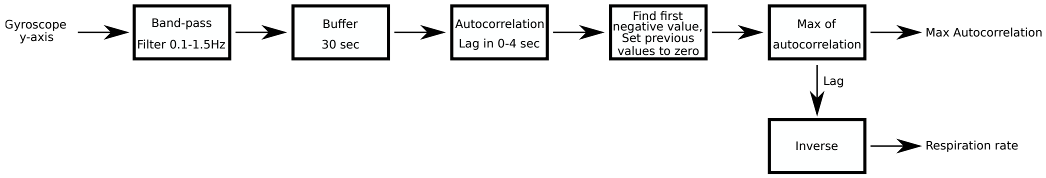

Figure 1: Flowshart of respiration autocorrelation algorithm.

Algorithm outline

1. Band-pass filter with second order Butterworth in 0.1-1.5Hz
2. Buffer into 30 sec non-overlapping windows and for each window calculate
   (a) Autocorrelation[1] function for lags $k \in [1, \text{sample rate} \times 4\ sec]$

   $$r_k = \frac{c_k}{c_0}, \tag{1}$$

   where c_0 is the sample variance of the time series and

   $$c_k = \frac{1}{T-1} \sum_{t=1}^{T-k} (y_t - \bar{y})(y_{t+k} - \bar{y}). \tag{2}$$

   Note that T in our case is (sample rate $\times$ 30 sec).

   (b) Find first l, where $r_l <= 0$. And set $r_p = 0$ with $p \in [1, l]$.

   (c) Find m, where

   $$m = \arg\max_k r_k \tag{3}$$

   (d) Output maximum autocorrelation value r_m and respiration frequency (min^{-1}) $f_{resp} = \frac{1}{m/\text{sample rate}} \times 60$

Note 1! Algorithm is simplified from Matlab version. The peak detection is removed and only maximum is taken from autocorrelation function.

[1]Refer to Matlab *autocorr* documentation for more information.

Activity Calculation

Jukka Ranta
Baba Center

September 15, 2020

Description

Movement activity is calculated from the accelerometer signal (sampling frequency 13 or 12.5 Hz) by taking a vector magnitude and band-pass filtering it. Then a rectified filter result is integrated over a moving window.

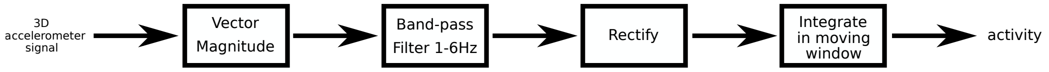

Figure 1: Flowchart for the movement activity calculation.

Algorithm outline

1. Calculate vector magnitude of 3D accelerometer sample;
$$m_t = \sqrt{x_t^2 + y_t^2 + z_t^2}. \quad (1)$$

2. Filter the magnitude signal with 1-6 Hz Butterworth band-pass filter. Output f_t.

3. Rectify filtered output
$$r_t = \sqrt{f_t^2} \quad (2)$$

4. Integrate in a moving window (5 sec) to get the activity
$$act_t = \sum_{T \in t \pm 2.5sec} r_T * \frac{1}{fs}, \quad (3)$$
where fs is sampling frequency.

Body Orientation Calculation

Jukka Ranta
Baba Center

September 15, 2020

Description

Body orientation is calculated from the gravity offset component in 3D accelerometer signal. The offset is actually the supporting force from the surface and points to the opposite direction of the gravity.

Orientation is divided into six different positions. Roll is defined as an angle on x-z - plane (transverse plane) and it can be used to determine the following orientations; left side, supine, right side and prone. Pitch is defined as an angle on y-z - plane (sagittal plane); head down, supine,head up and prone. The plane of the largest combined gravity vector is chosen for the final position.

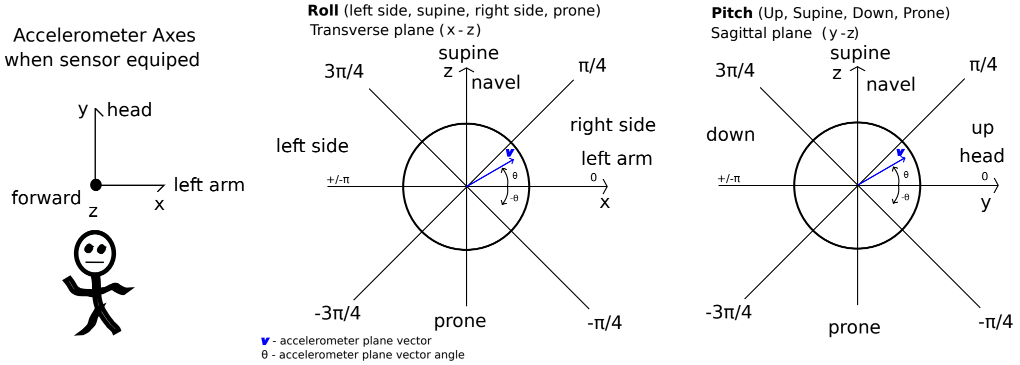

Figure 1: Illustration of the planes for orientation determination.

Algorithm outline

1. Smooth each accelerometer signal channel. In Matlab 10s median filter was used.

2. For each filtered sample calculate

   (a) Vector magnitude in both planes separately

   (b) Angle using atan2-function (four-quadrant inverse tangent) in both planes. Returns the angle in $\pm\pi$.

   (c) Determine the quadrant using angle thresholds (see figure above) in both planes. Code into body position.

   (d) Select the plane which has larger vector magnitude for representative plane and output its position.

Note 1! For smoothing moving average is ok. There was a discussion to use shorter 5 sec window.
Note 2! Use the following position coding:

```
Roll is defined as an angle on x-z - plane;
(left side, supine, right side, prone) coded to (1,2,3,4).

Pitch is defined as an angle on y-z - plane;
(down,supine,up,prone) coded to (5,2,6,4).
```

Note 3! You can do the plane selection (2.d) directly after magnitude calculation (2.a) so you don't need to calculate the angle in both planes.
