# Supplementary material for: NAPping PAnts (NAPPA): An open wearable solution for monitoring Infant's sleeping rhythms, respiration and posture
Source: Heliyon. 2024 Jun 21;10(13):e33295. doi: 10.1016/j.heliyon.2024.e33295 (PMC11255670; doi:10.1016/j.heliyon.2024.e33295)
Supplement: Multimedia component 4 [file mmc4.docx]

**SUPPLEMENTAL MATERIAL**

1. In-Sensor Feature Logging
2. Example Report *(Please review the individual supplementary materials.)*
3. Classifier Result *(Please review the individual supplementary materials.)*
4. Comparison Between Alternative Classifier Targets
5. Features *(Please review the individual supplementary materials.)*
6. Description of Normalisation techniques
7. Age Distribution of the Infants
8. Accounting for the Age-Related Change in Respiration Rate and Other Features
9. Accounting for Class Imbalance in Sleep State Classification
10. Individual Level Results
11. User Experience Questionnaire
12. Results of Training the Classifier with Subject-Wise Normalisation
13. Classifier Result CSV: Column Definitions and Explanations
14. References

**In-Sensor Feature Logging (S1)**

For allowing NAPPA recordings without real-time data streaming, the feature computation was implemented in C++ code, and the feature values were logged into the sensor's EEPROM memory along with the timestamp, which was automatically synchronised with universal (UNIX) time via the mobile application. Features were coded using Movesense Device SDK written in C++ language, and their correspondence to Matlab implementation was verified using a sensor simulator (part of the Movesense SDK package), and a direct comparison of feature values computed independently in the C++ code vs Matlab scripts. The observed near-complete equivalence supported a continuation of NAPPA recordings with the in-sensor feature logging only.

The feature calculations were initially developed and tested using Matlab scripts, so their implementation in the C++ code in the programmable sensor required validation by verifying correspondence of the two approaches. Here, we show correspondence of the feature values obtained by Matlab (both Offline and Online) and Movesense simulator in time series and scatter plots (see below). Note that 2D plots for Online Matlab implementation and C++ code are slightly shifted in feature values to avoid the plots overlapping.


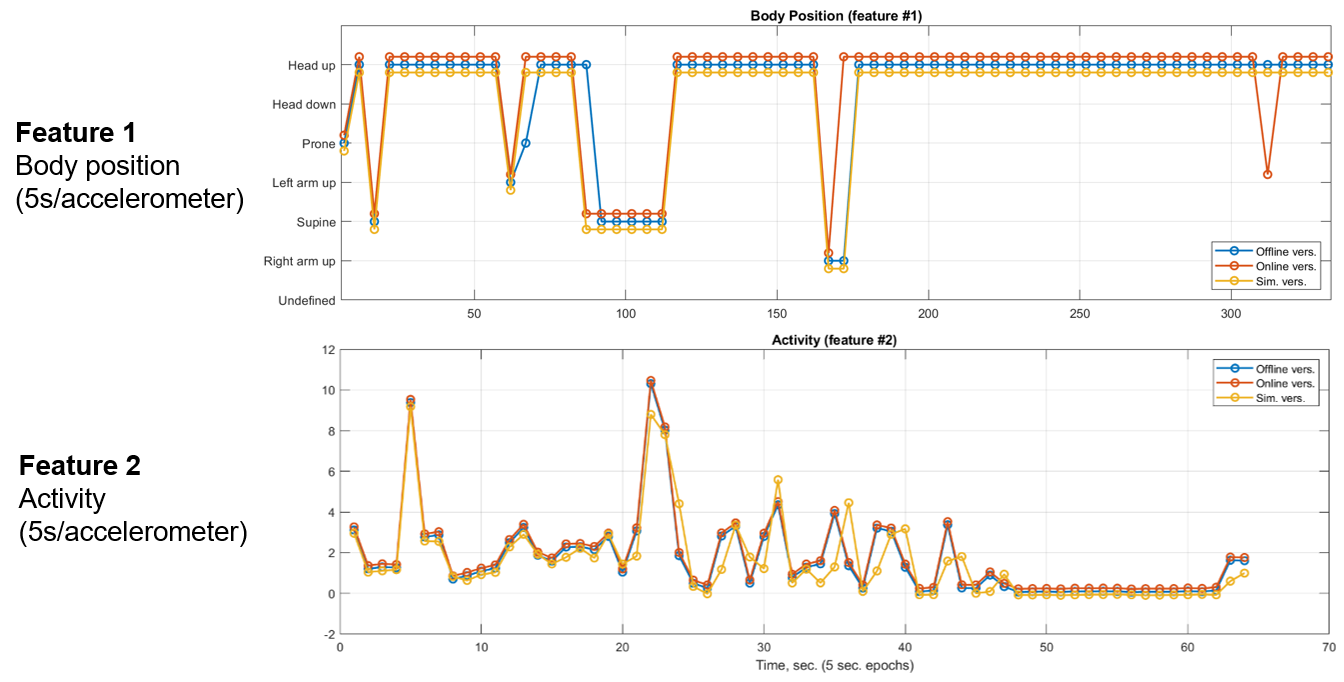


**
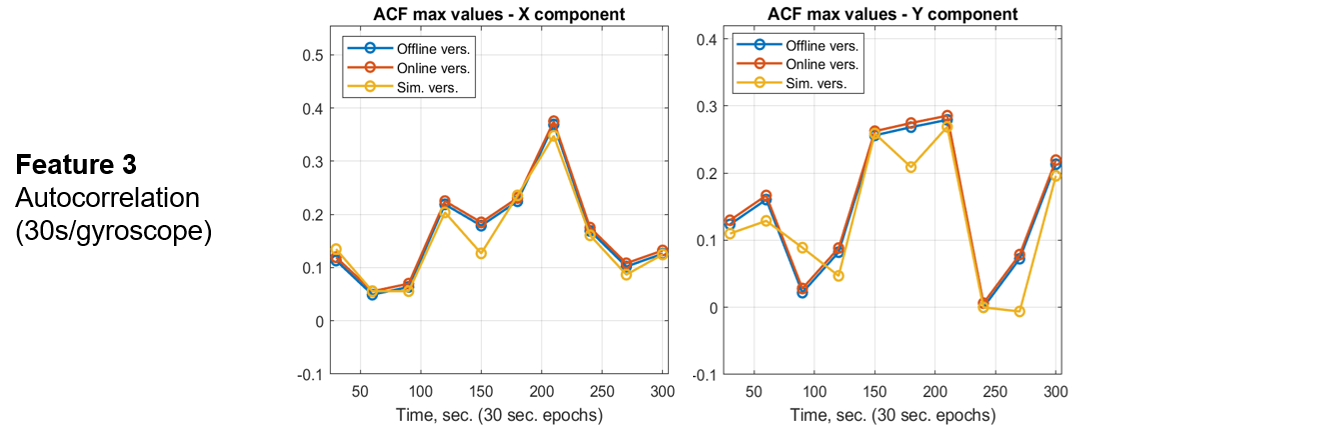
**

**
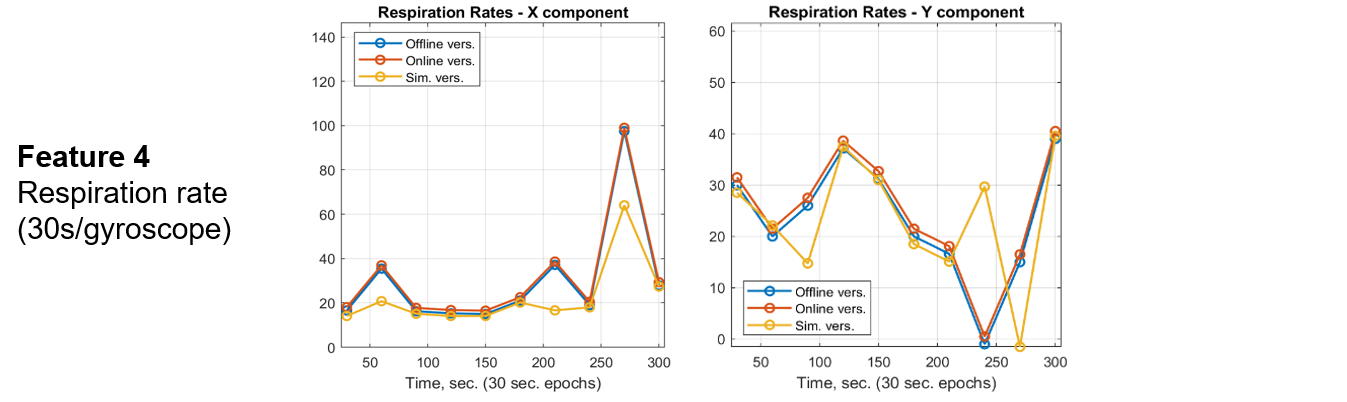
**

**
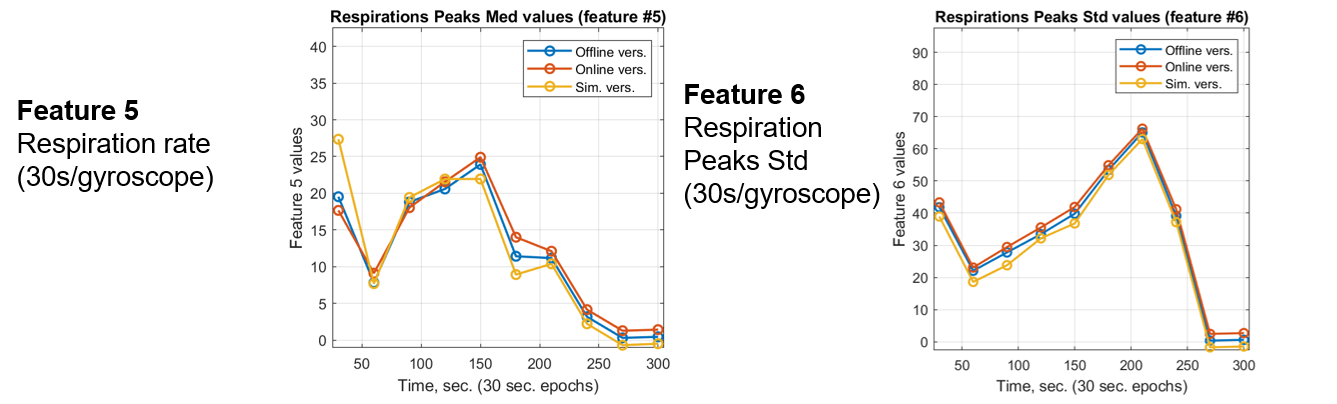
**


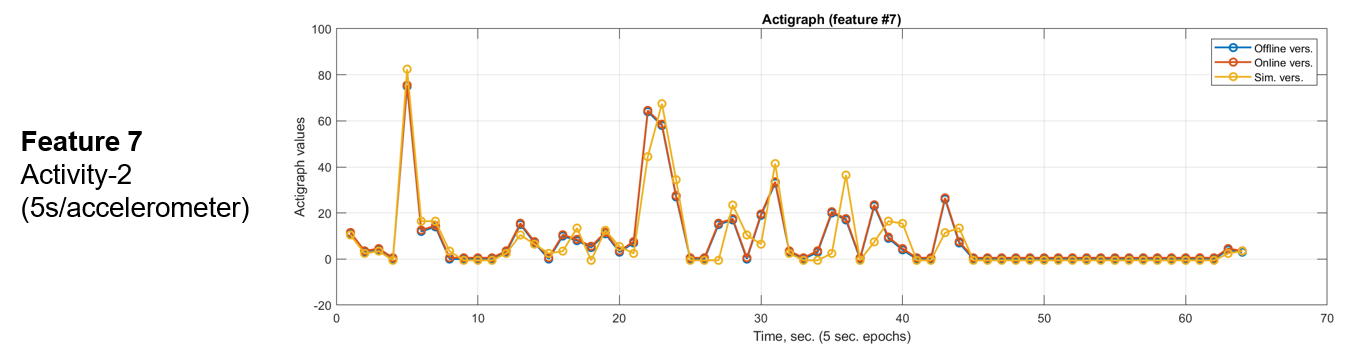


An additional data logger (NAPPA logger) was also built with an intuitive and easy-to-use interface for starting and stopping the sensor firmware as well as data download from the sensor and further upload to a desired location. The firmware was programmed to pack all recordings into a single compressed (.zip) file for easier handling.

**Comparison Between Alternative Classifier Targets (S4)**

Alternative classifier targets were compared to assess how well the NAPPA data can distinguish between different groupings of sleep states. The training data used in the supplements was the same as that used for the classifier reported in the main paper, enabling a direct comparison of performance measures.

The confusion matrices display classifier performance for the 3-state detection of wake/REM/NREM (left) and for the full 5-state detection of wake/REM/N1/N2/N3 (right). The overall detection performance is clearly lower in both cases compared to the classifier proposed for NAPPA use. Additionally, a detailed inspection shows that: 1) the given target classes show the best detection, except for N1-detection in the 5-state model. 2) However, there is a substantial leak of detection to the neighbouring sleep states, most prominently for REM and N1, but also between N2 and N3.


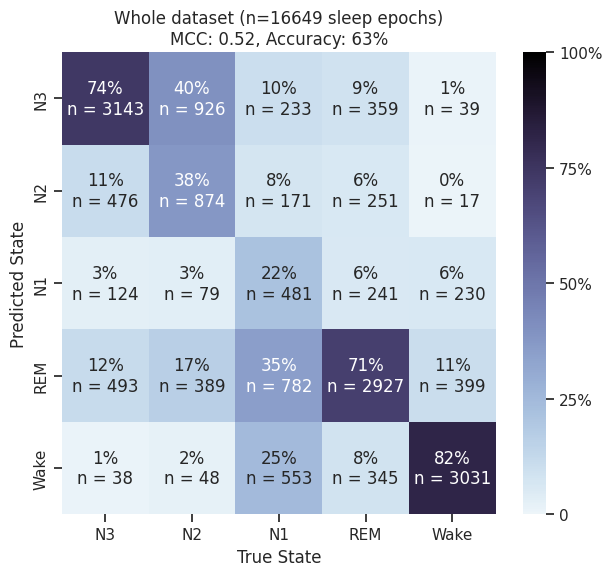

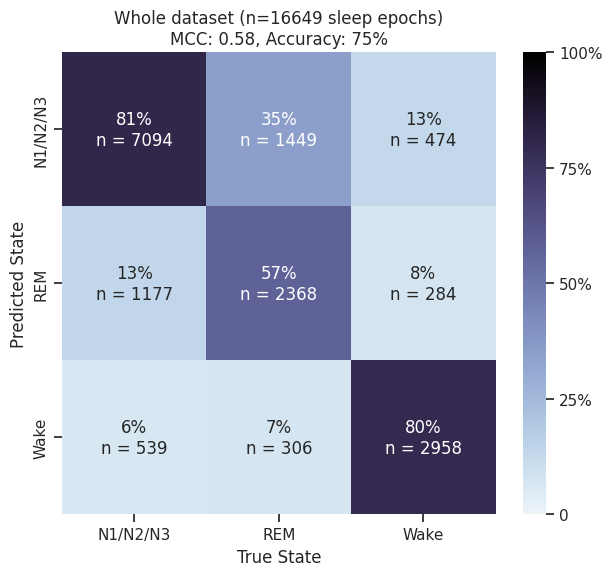


We also evaluated the theoretical potential of classifiers to utilize NAPPA data for these detection tasks by projecting the high-dimensional feature space into a reduced 2-dimensional space using the established t-SNE visualization (lower figures). These figures clearly demonstrate that the separability between states is strongly reduced with a higher number of states (left side), while this 3-state model (wake/REM/NREM) already exhibits substantial overlap of N1/REM with the other classes.


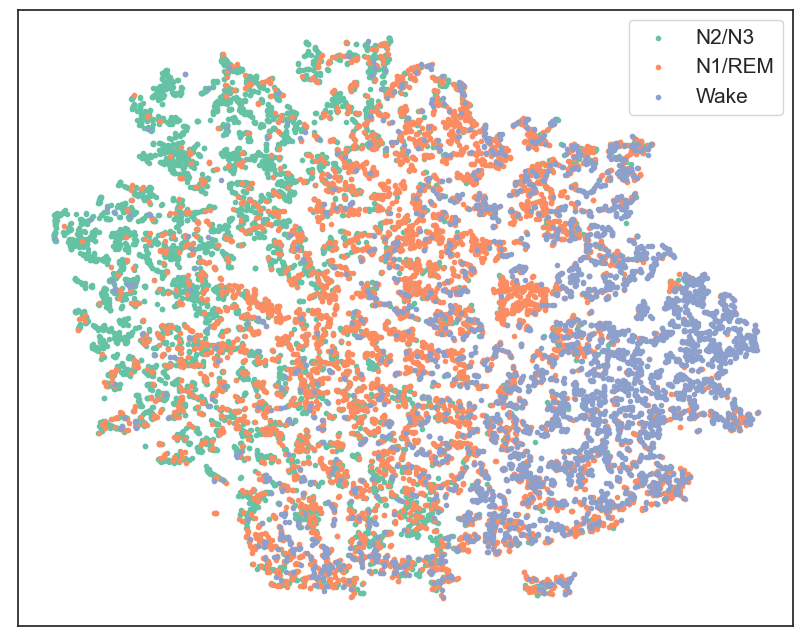

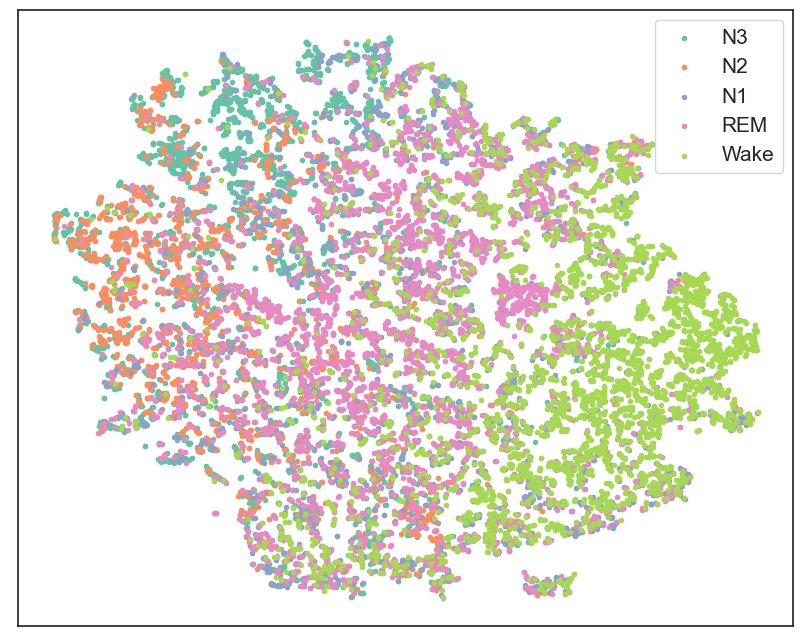


Taken together, these data-driven experiments support the physiologically driven reasoning that movement recording alone is not optimal for the detection of REM or N1 sleep.

**Description of Normalisation Techniques (S6)**

The normalisation of features was carried out using a hybrid approach. Heuristic scaling was applied to the activity and respiration autocorrelation features, while global or subject-wise z-score normalisation was employed for the remaining features. To scale the activity and respiration autocorrelation features, we chose to apply dataset-independent, predetermined heuristic transformations rather than the z-score normalisation, which relies on the descriptive statistics (mean, standard deviation) of the features in the dataset. The following piecewise function is used to scale the activity feature:


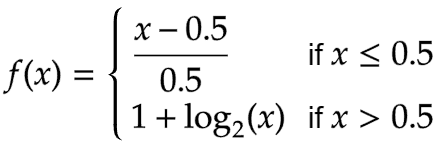


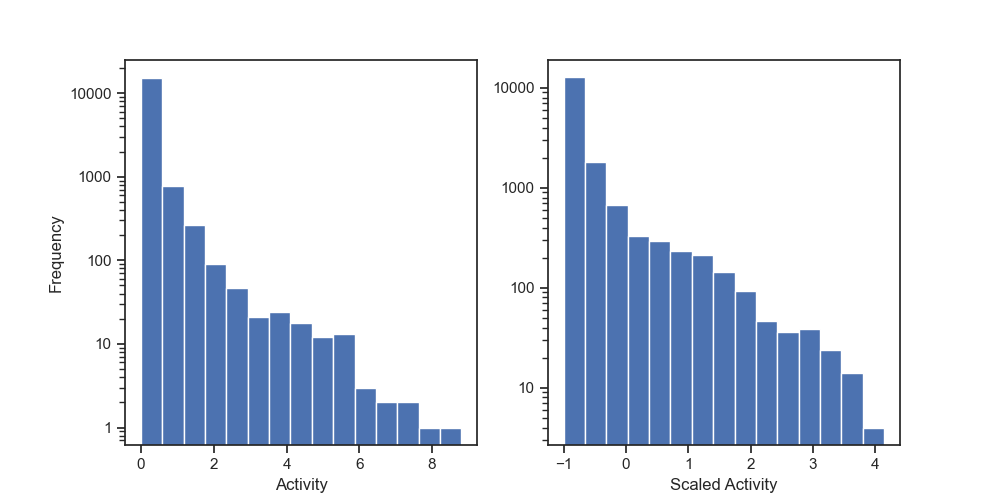


Left: The original distribution of the activity feature data. Right: Post-transformation, the tail of the distribution is shifted left of zero and large values are scaled down. Notice that we used logarithmic scale in the y-axes of the histograms due to the nature of the distribution.

By shifting the tail of the distribution to the left of zero, we bring the values to a similar range as the z-score normalised features while keeping the mean close to zero. Additionally, the choice of a logarithmic transformation for values greater than 0.5 effectively scales down larger values, compacting the tail.

For scaling the respiration autocorrelation feature, we used the following function:


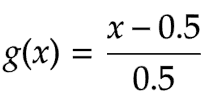


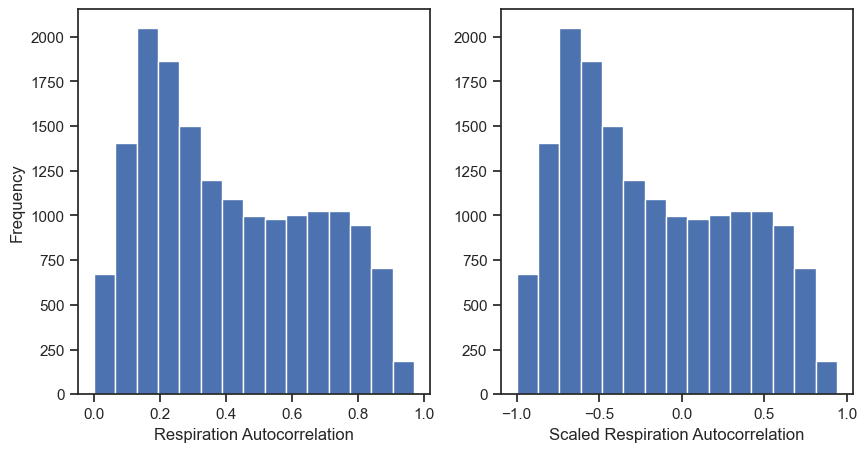


Left: The original distribution of the respiration autocorrelation feature. Right: The distribution after applying the g(x) transformation.

This transformation effectively realigns the mean of the distribution closer to zero and brings the values to a similar range with the z-score normalised features.

**Age Distribution of Infants (S7)**

Below, you can see the age distribution of infants ranging from 2 weeks to 18 months of age with a median age of 3 months (IQR: 1-4 mos).


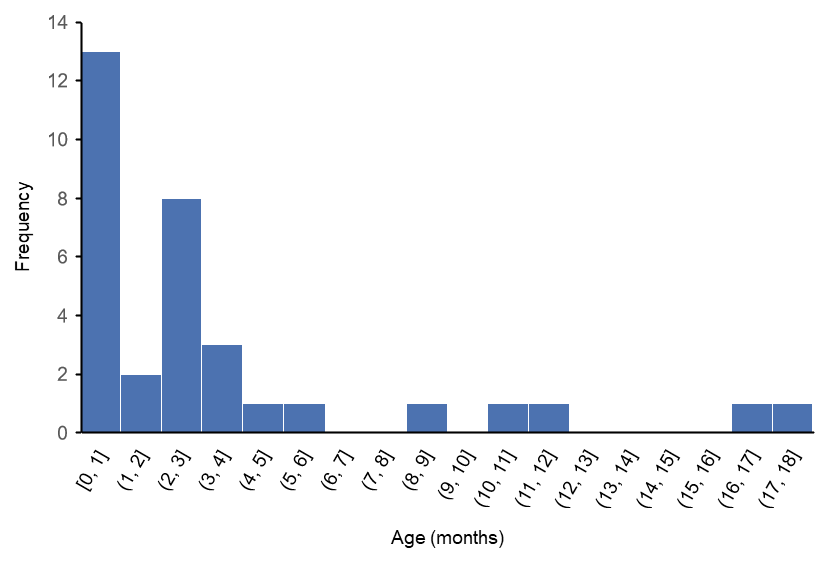


**Accounting for the Age-Related Change in Respiration Rate and Other Features (S8)**

Likely, at least some of the computational features used in our work are age-dependent, potentially confounding the classifier performance. Therefore, we systematically assessed the age-dependency of these features using all the NAPPA recordings available from the cohort described in this study, as well as those from ongoing clinical trials conducted at homes (total N= 178). The scatter plots below show that only respiration rate was significantly correlated to the age, while other features showed none or practically meaningless correlation.


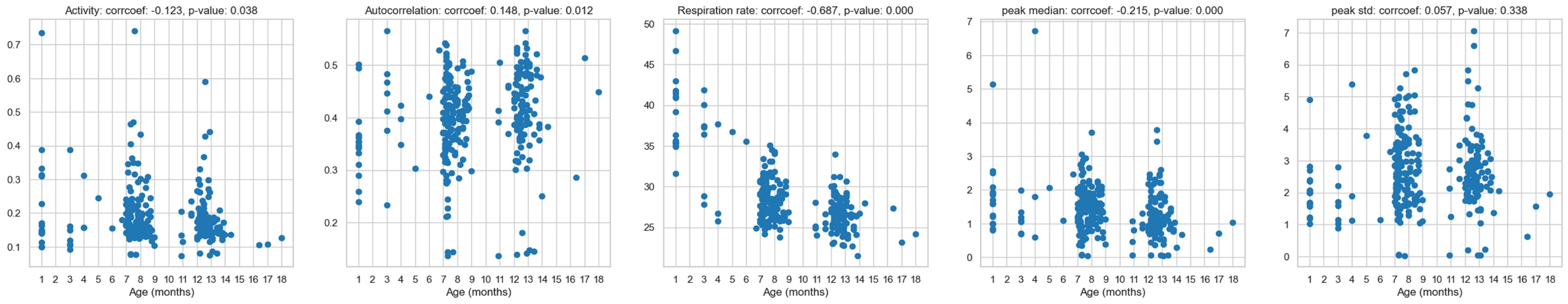

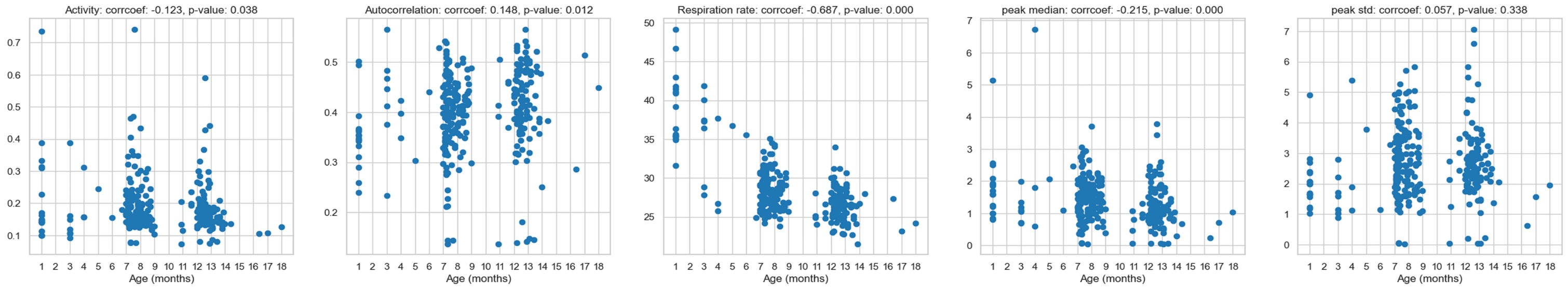


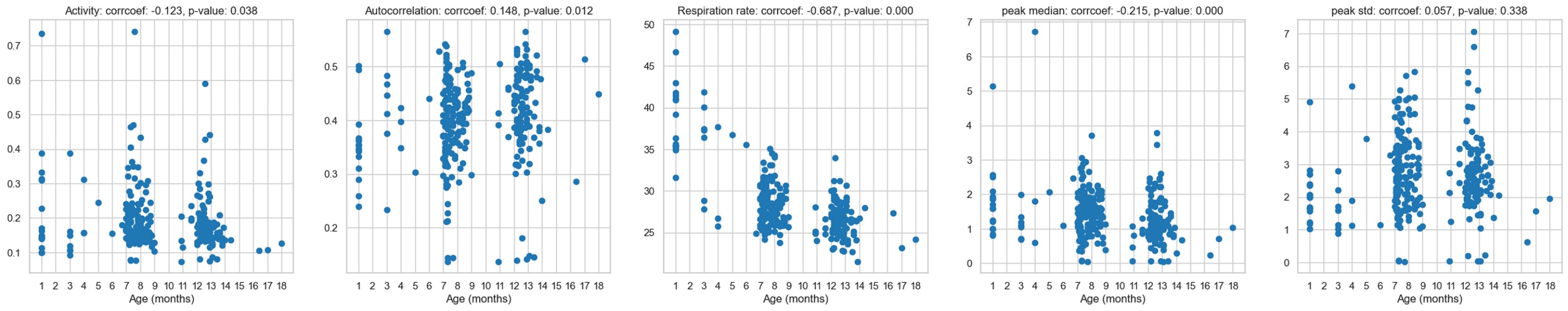

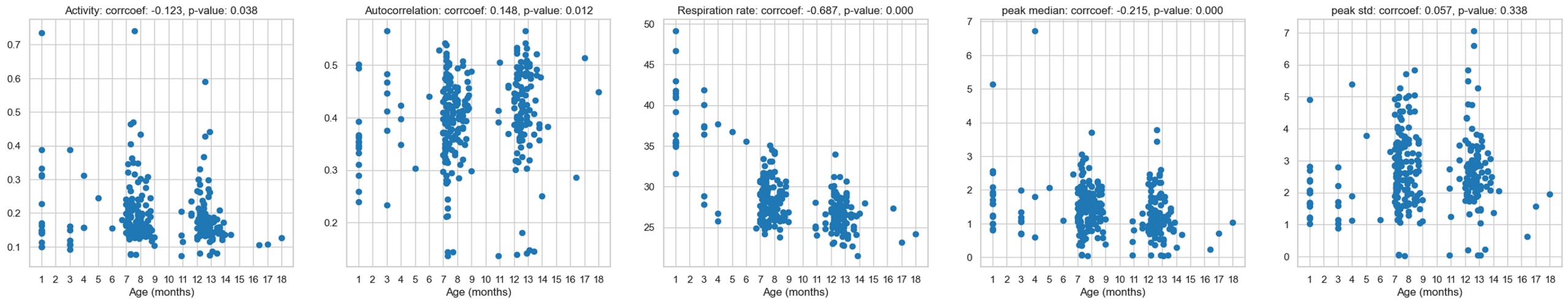

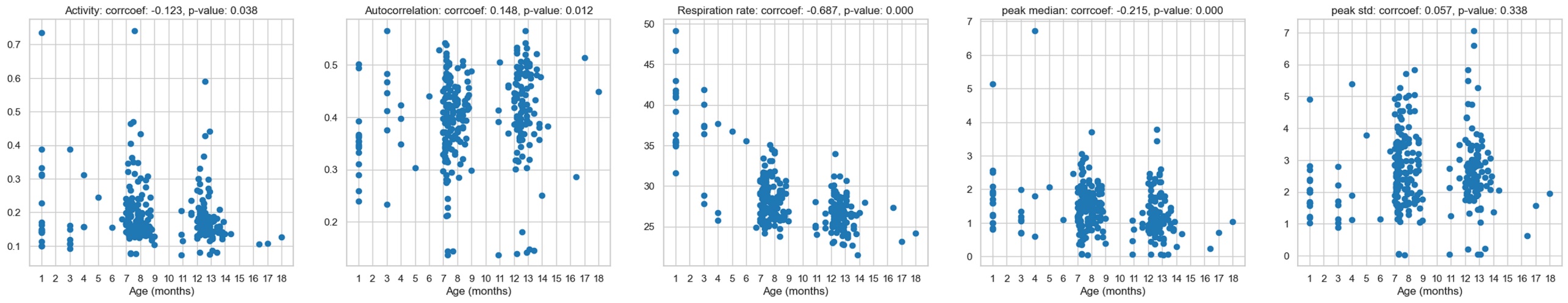


The scatter plots in the top row show no change in activity (left) and a significant decline in respiration rate with age (right). The scatter plots in the bottom row show low or non-significant age correlations for the respiration features: *peak median* (left), *peak standard deviation* (middle), or *autocorrelation* (right).

Based on these observations, we then used our present training dataset and investigated two strategies to address the observed correlation (*r* = -.687, *p* = 0.000) between respiration rate and age in our dataset. First, we explored whether embedding age as a separate feature could enhance the model performance. Second, we used linear regression to estimate the relationship between the mean respiration rate and age. Using the obtained linear fit, we mapped each subject's age to a corresponding mean respiration rate value. Subsequently, we adjusted the respiration rate data for each sleep recording by subtracting the estimated mean respiration rate value (based on age) from the actual respiration rate values to eliminate the correlation. However, neither of the two approaches helped improve the classification performance hence they were left out of the final model.

**Accounting for Class Imbalance in Sleep State Classification (S9)**

We studied whether assigning weights inversely proportional to the frequency of each sleep class could be utilised to mitigate the imbalance of sleep states in our dataset. The histogram below visualises the underrepresentation of the wake class, while the classes deep sleep (N3/N2) and light sleep (N1/REM) have roughly equal representations.


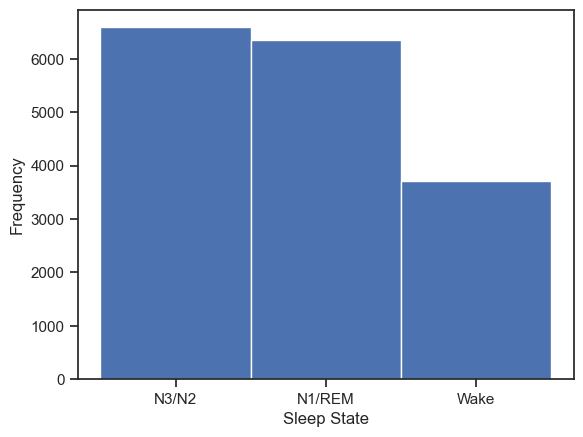


The class weights are employed during the loss computation in the training process, penalizing the misclassification of the underrepresented ‘Wake’ class with a higher cost for the model. In each LOSOCV iteration, we computed the class weights from the training data using the compute_class_weights function from the scikit-learn^1^ package. This function provides the weight for each class based on the following formula:


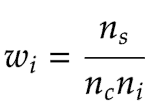


Where $w_{i}$ is the weight for class $i$ , $n_{s}$ is the total number of data points, $n_{c}$ is the number of classes, and $n_{i}$ is the number of data points in class $i$.

Applying this technique significantly improved the classification accuracy of the wake class significantly, as expected. However, for an unknown reason, in turn weakened the classification accuracy of the light sleep class. Consequently, it did not provide an overall performance improvement and was thus excluded from the final model.


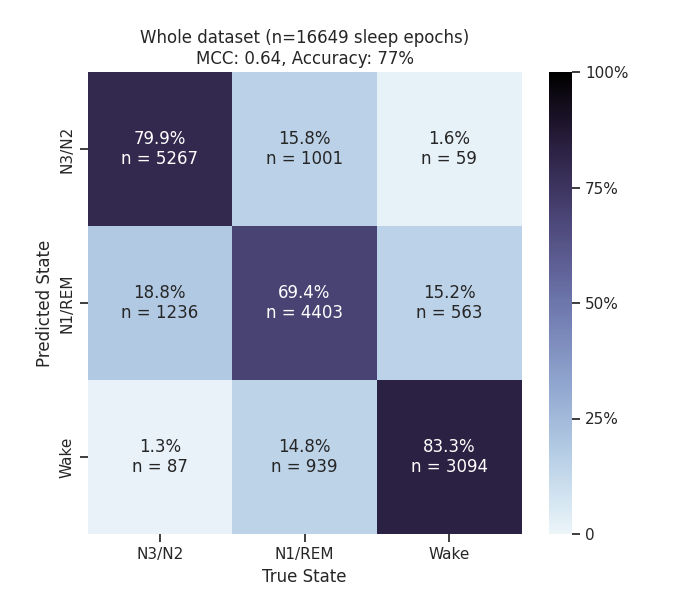


This figure illustrates the group-level results after applying class weighting during the training phase. Wake class is now identified with the highest accuracy, while the accuracy of detecting light sleep is decreased. The change in the accuracy of detecting deep sleep is minimal. It is important to note that the overall Matthews Correlation Coefficient (MCC) score and accuracy remain unchanged when compared to the results of the final model in the main paper.

**Individual Level Results (S10)**

Individual-level results are presented below using both confusion matrices and Sleep Depth Trend (SDT) outputs. The first table displays results using global normalisation (the method of choice reported in the main paper), while the second table shows results using subject-level normalization (alternative method).

| Individual-level results with the global normalization (method of choice) | | | |
| --- | --- | --- | --- |
| *Subject number* | *Age* | *HYPNO+SDT plot* | *Confusion matrix* |
| *1* | *2 mos* | *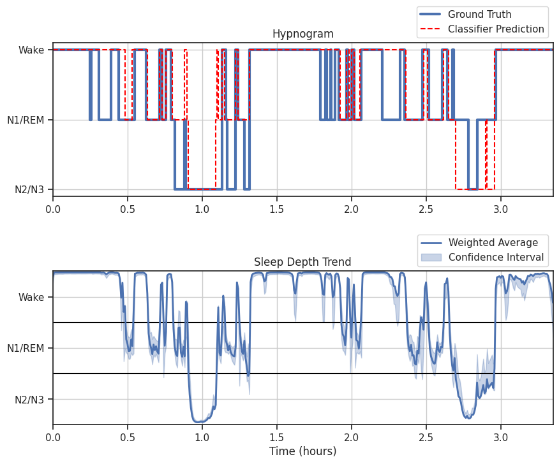* | *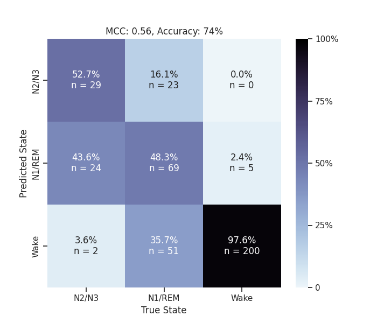* |
| *2* | *1 mos* | *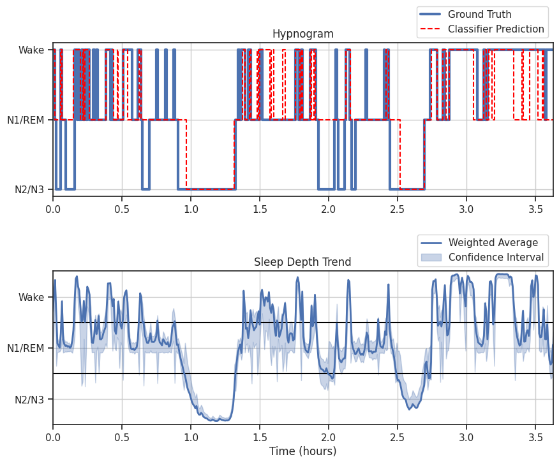* | *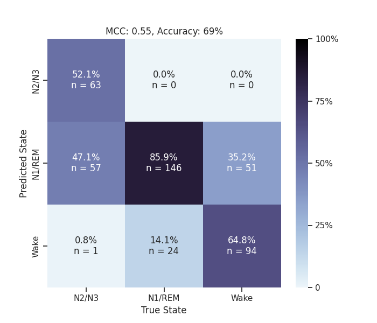* |
| *3* | *2 wks* | *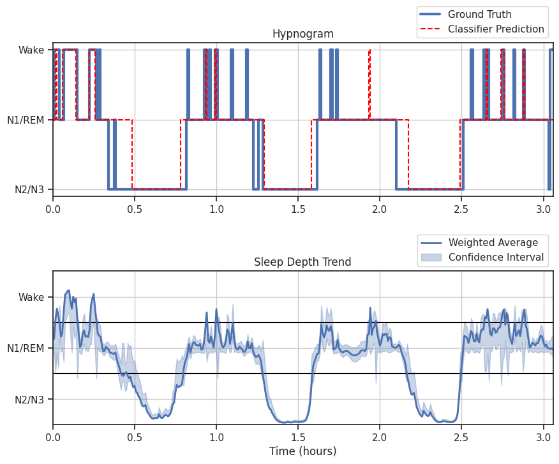* | *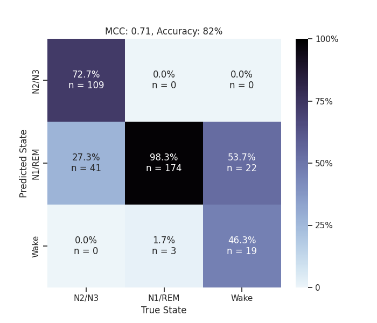* |
| *4* | *7 wks* | *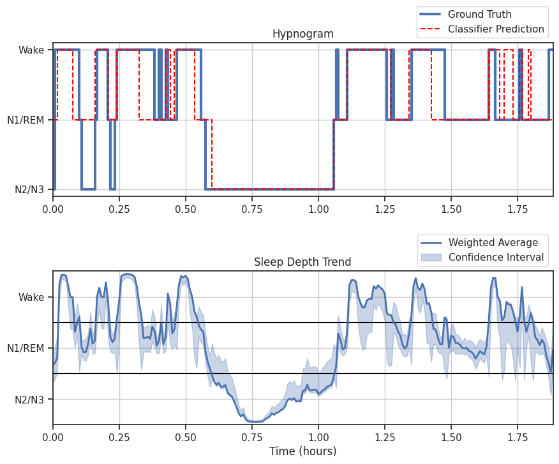* | *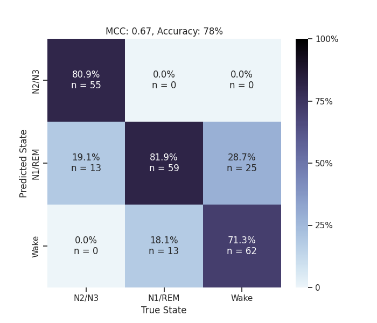* |
| *5* | *1 wk* | *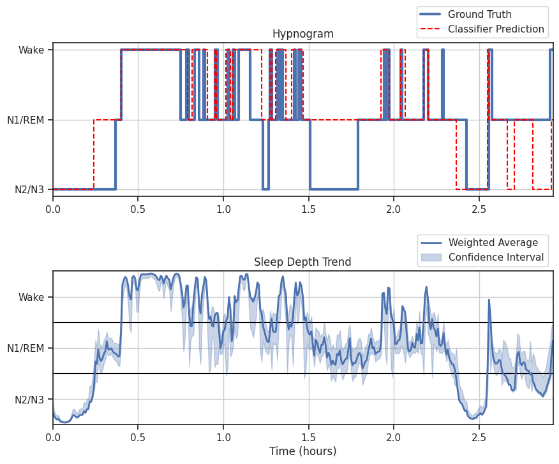* | *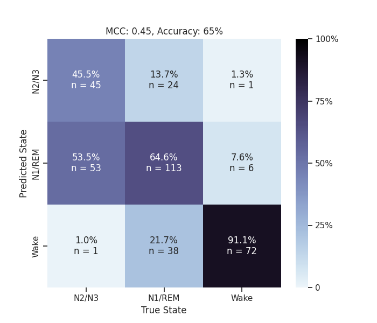* |
| *6* | *1 mos* | *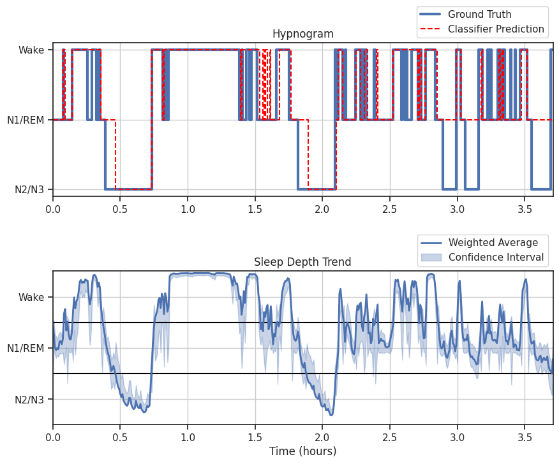* | *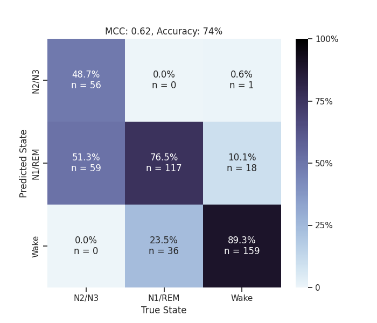* |
| *7* | *3 mos* | *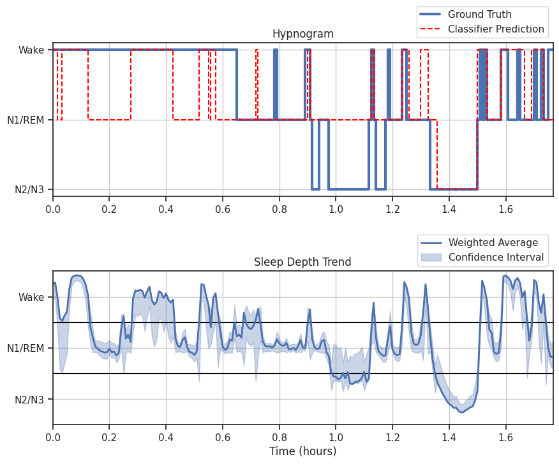* | *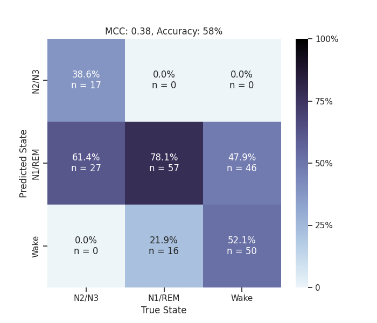* |
| *8* | *1 mos* | *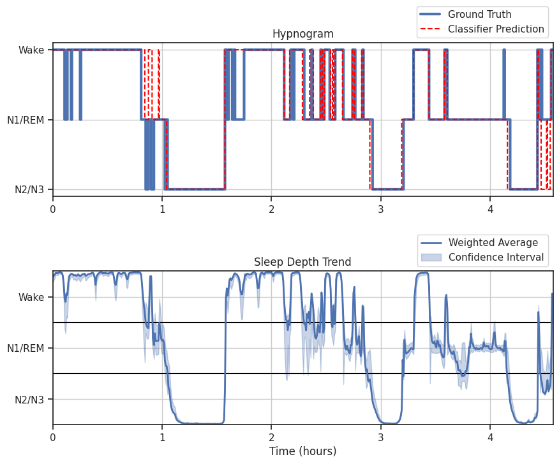* | *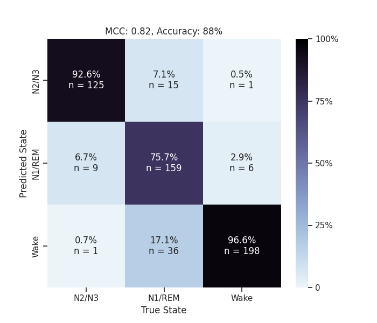* |
| *9* | *3 wks* | *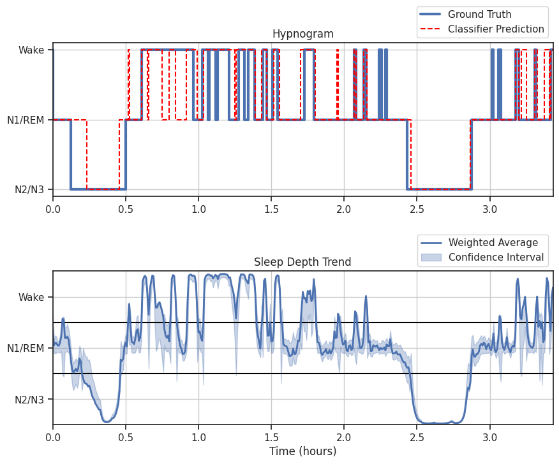* | *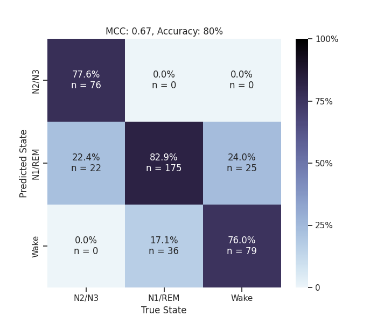* |
| *10* | *1 mos* | *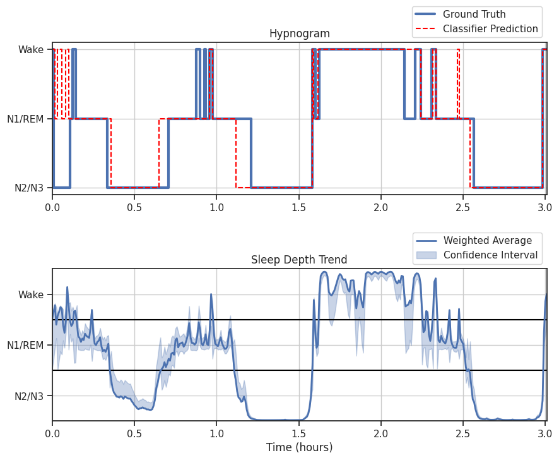* | *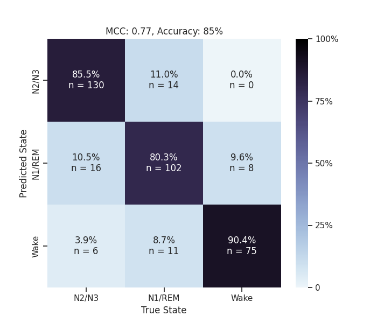* |
| *11* | *1 mos* | *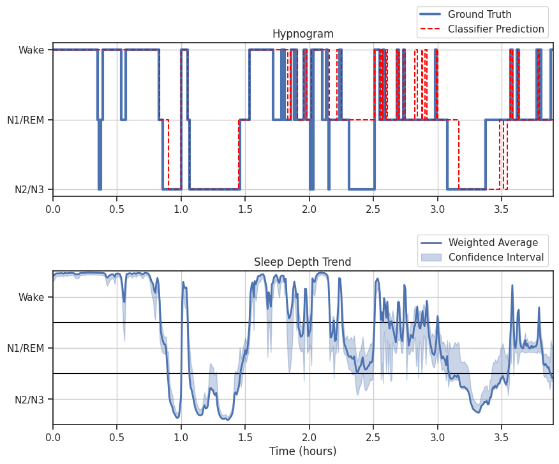* | *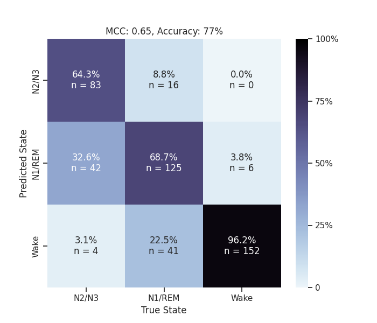* |
| *12* | *5 mos* | *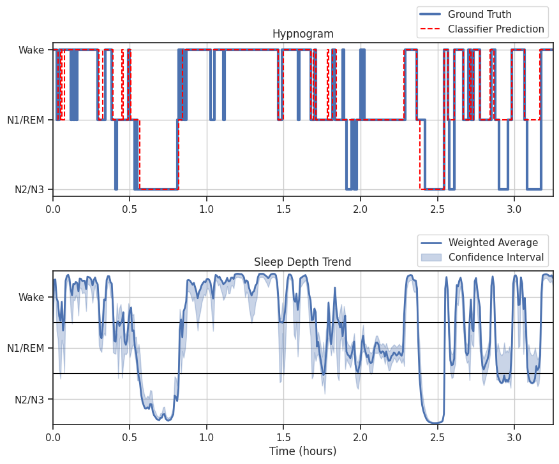* | *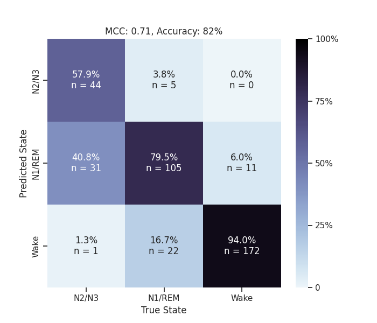* |
| *13* | *4 mos* | *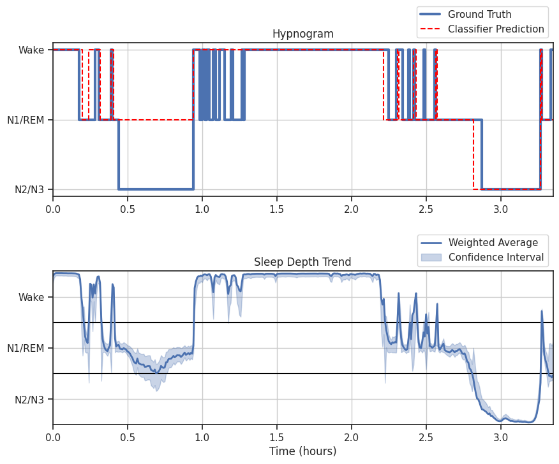* | *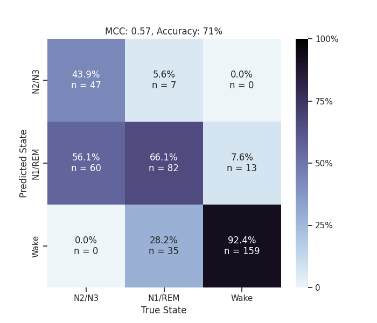* |
| *14* | *6 mos* | *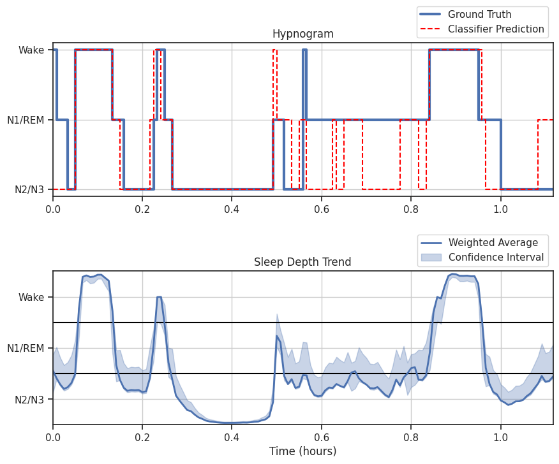* | *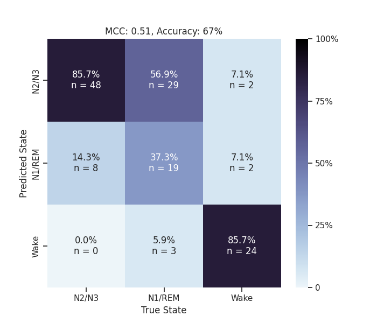* |
| *15* | *4 mos* | *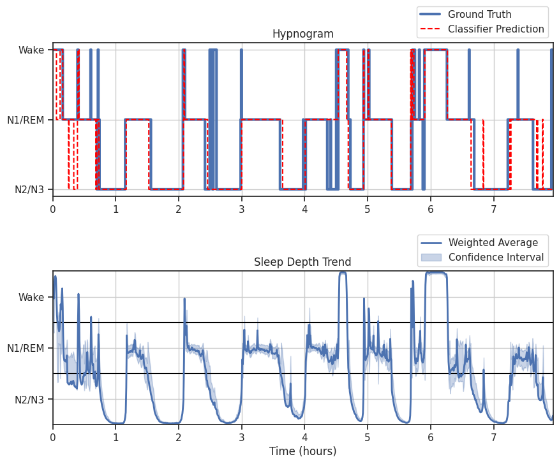* | *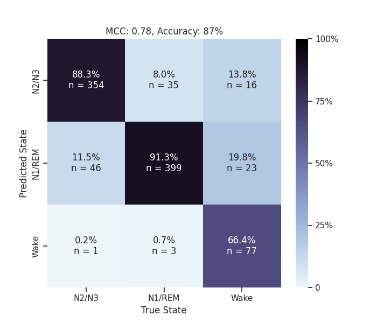* |
| *16* | *1 mos* | *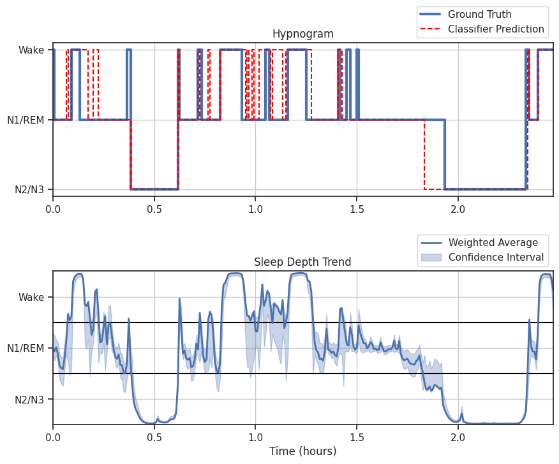* | *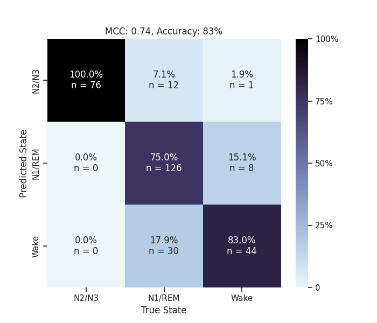* |
| *17* | *3 mos* | *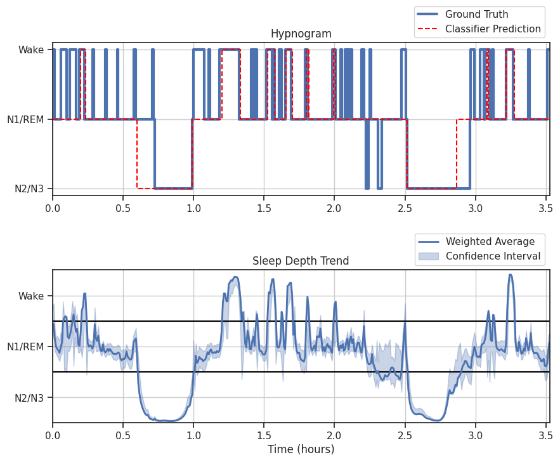* | *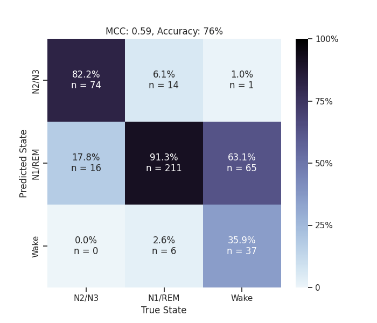* |
| *18* | *3 mos* | *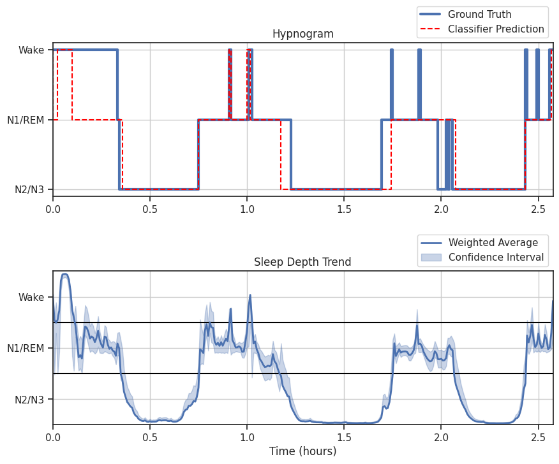* | *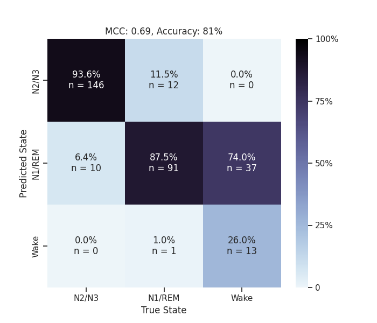* |
| *19* | *3 mos* | *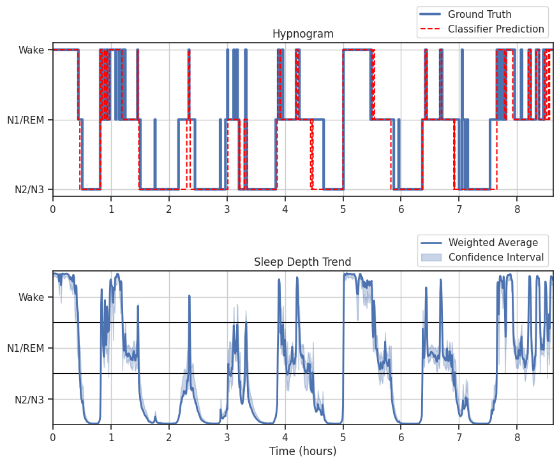* | ***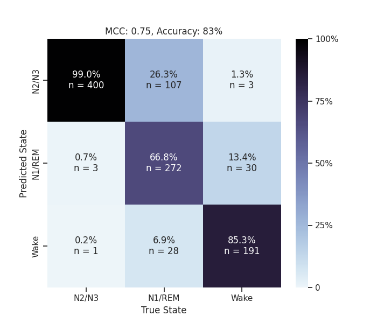*** |
| *20* | *9 mos* | *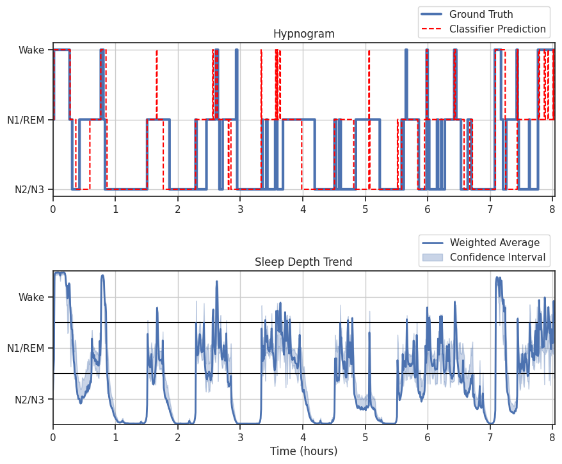* | ***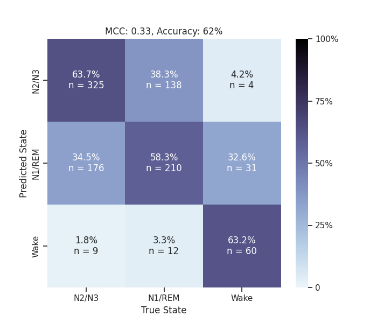*** |
| *21* | *1 mos* | *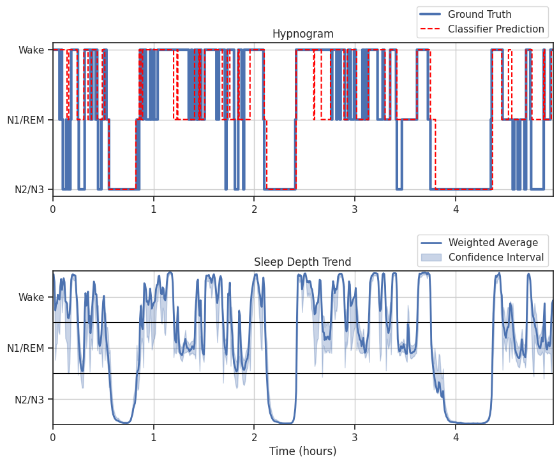* | ***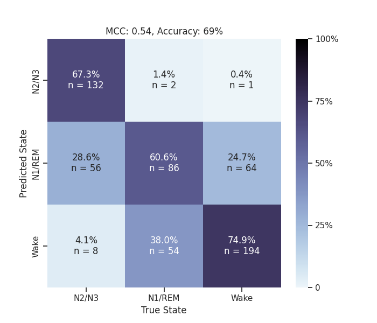*** |
| *22* | *3 mos* | *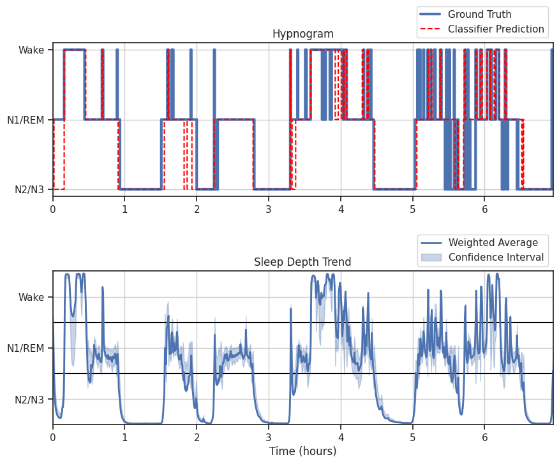* | ***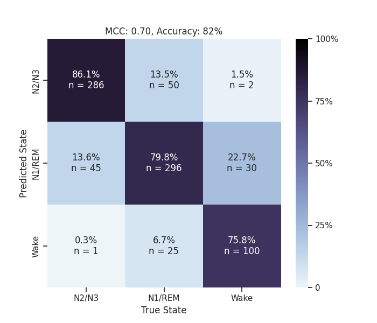*** |
| *23* | *1 mos* | *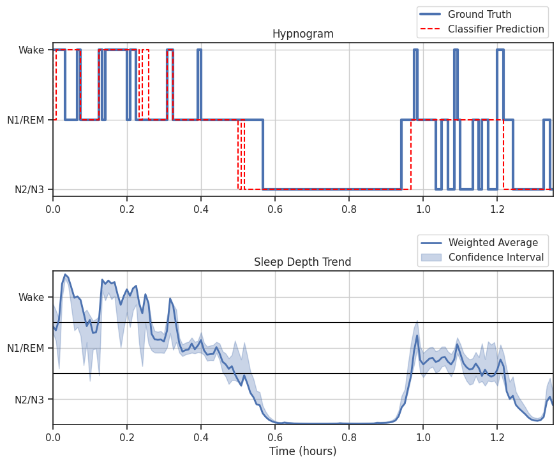* | ***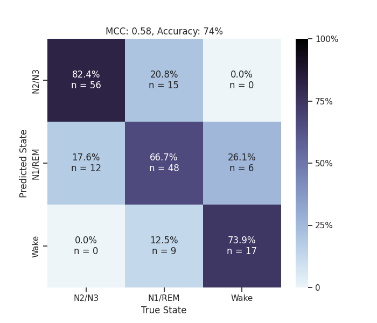*** |
| *24* | *3 mos* | *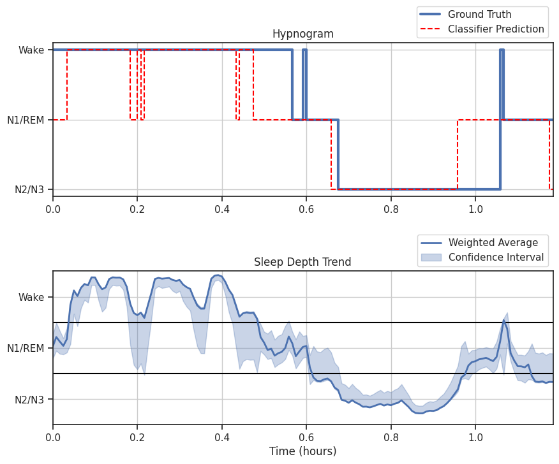* | ***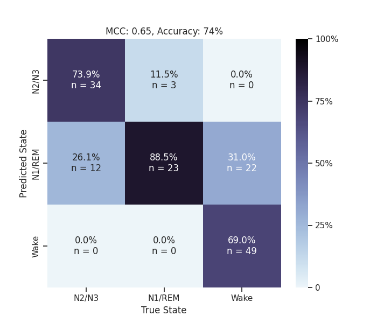*** |
| *25* | *1 mos* | *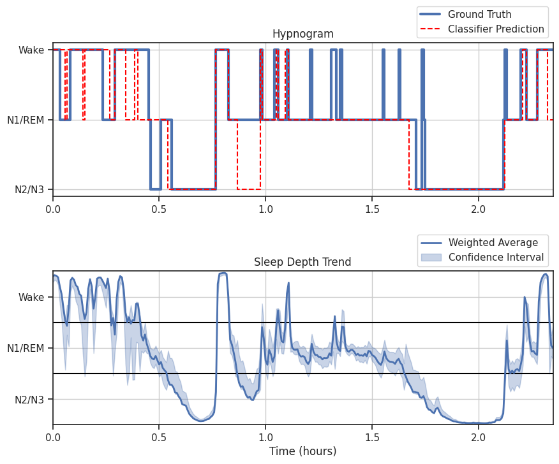* | ***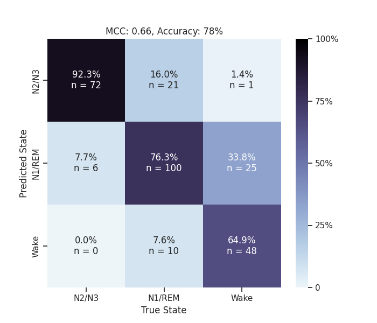*** |
| *26* | *3 mos* | *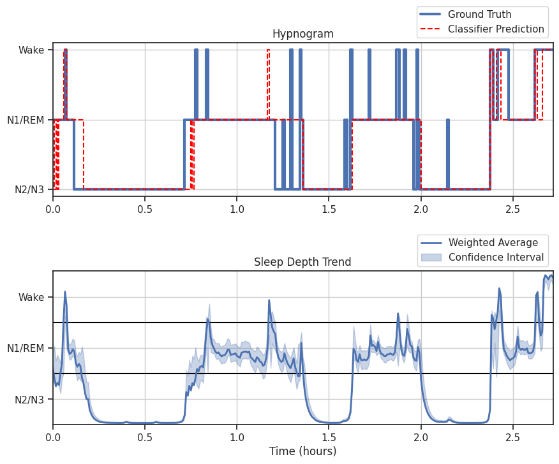* | ***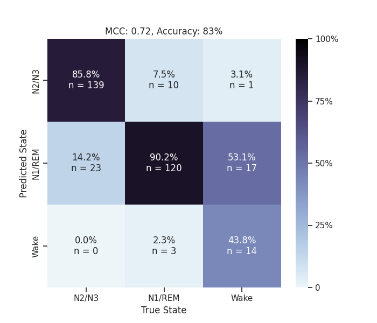*** |
| *27* | *4 mos* | *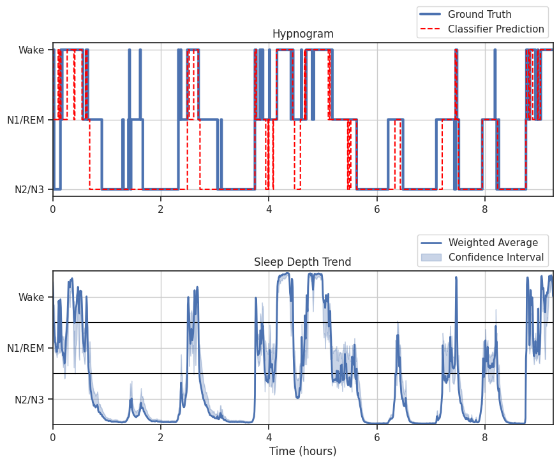* | ***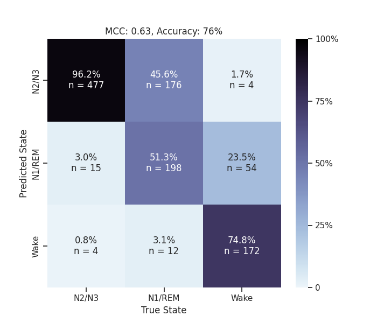*** |
| *28* | *12 mos* | *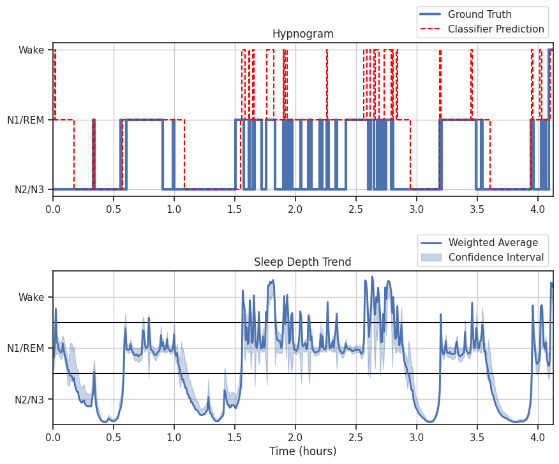* | ***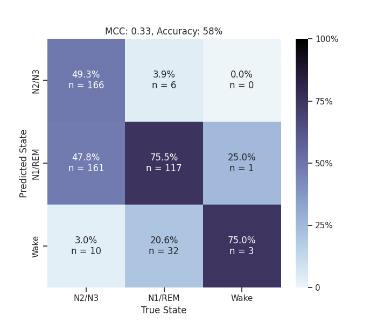*** |
| *29* | *3 mos* | *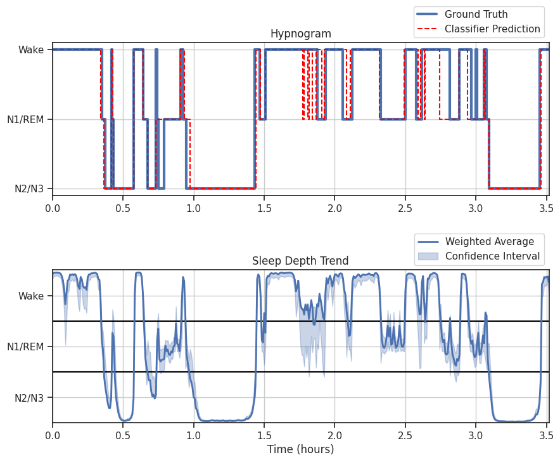* | ***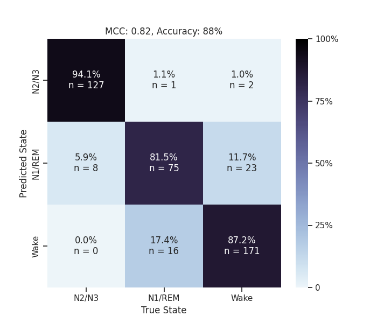*** |
| *30* | *1 mos* | *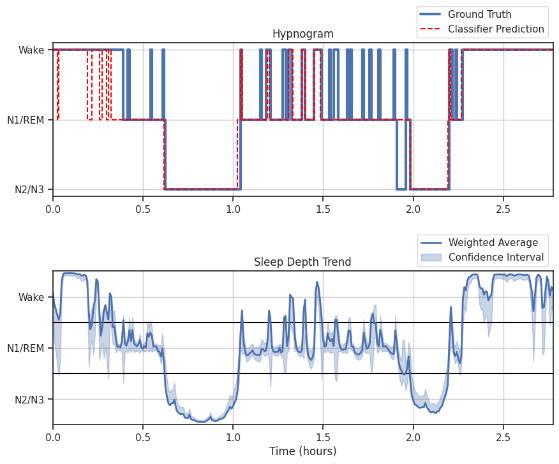* | ***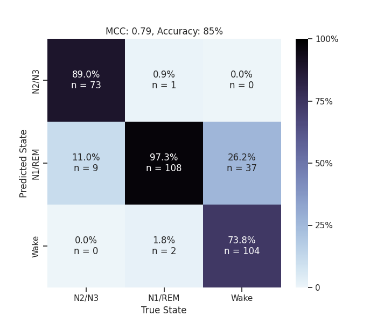*** |
| *31* | *17 mos* | *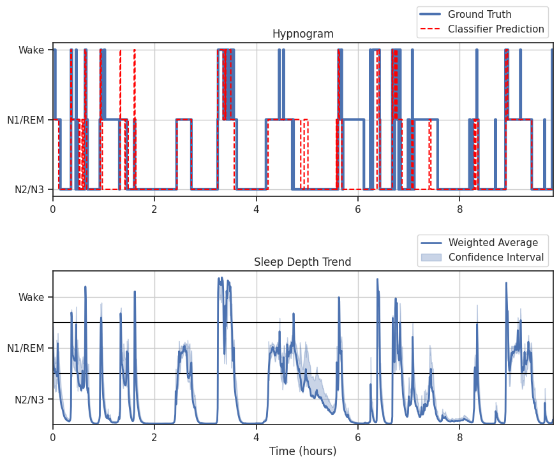* | ***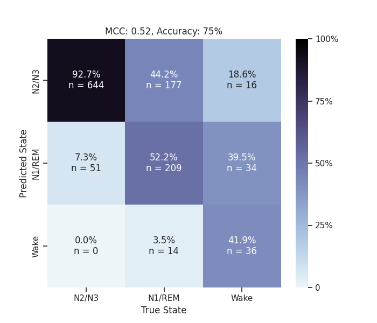*** |
| *32* | *18 mos* | *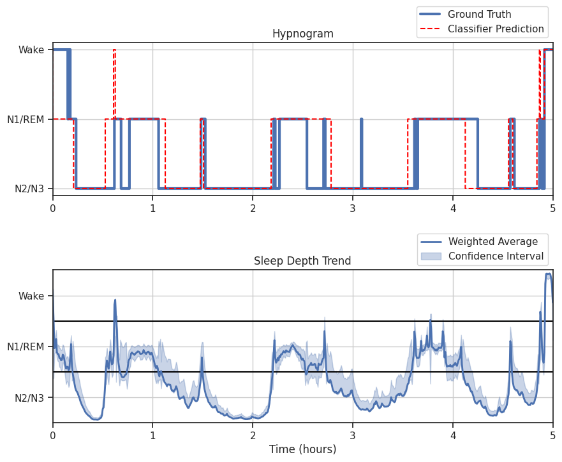* | ***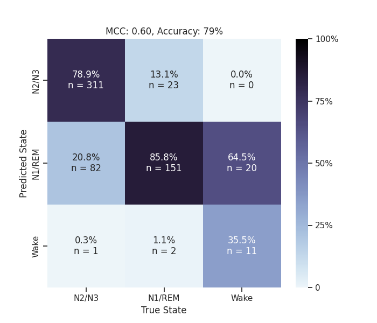*** |
| *33* | *11 mos* | *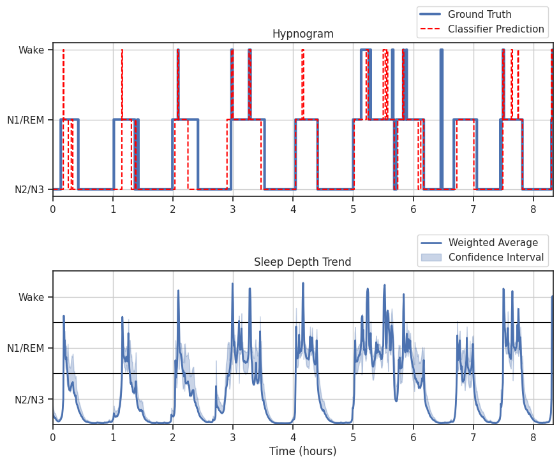* | ***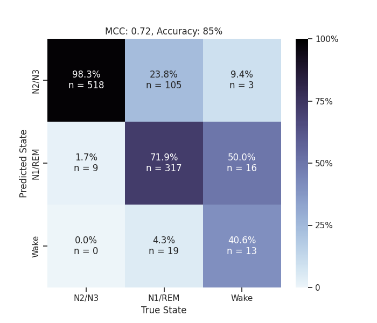*** |

| Individual-level results with the subject-level-normalization (method of choice) | | | |
| --- | --- | --- | --- |
| *Subject number* | *Age* | *HYPNO+SDT plot* | *Confusion matrix* |
| *1* | *2 mos* | *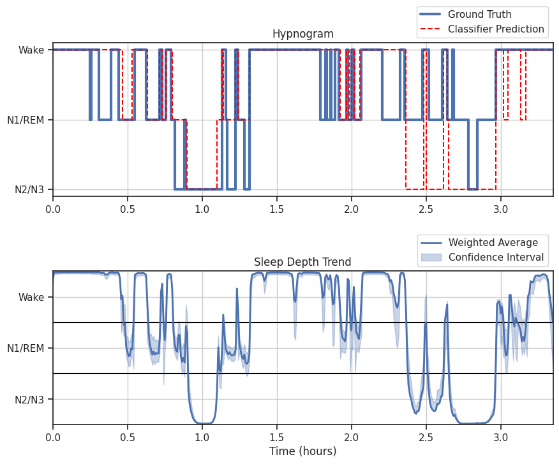* | *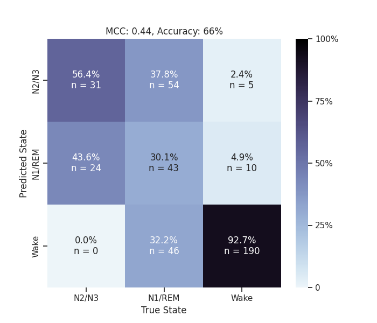* |
| *2* | *1 mos* | *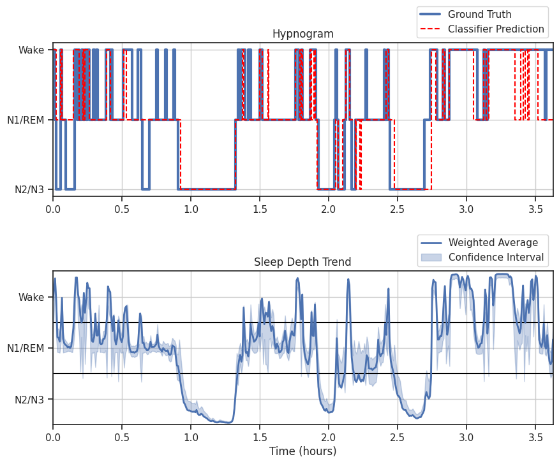* | *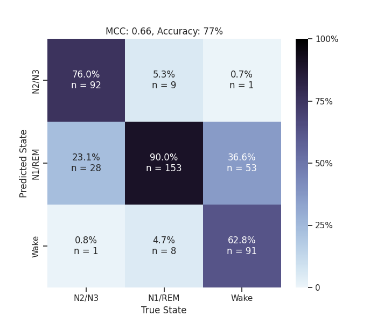* |
| *3* | *2 wks* | *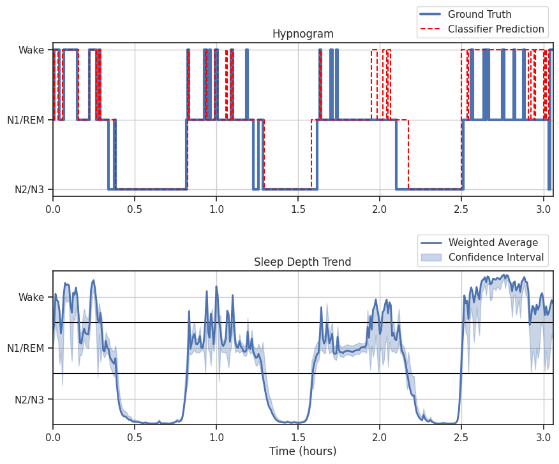* | *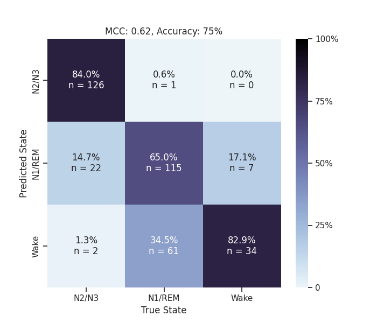* |
| *4* | *7 wks* | *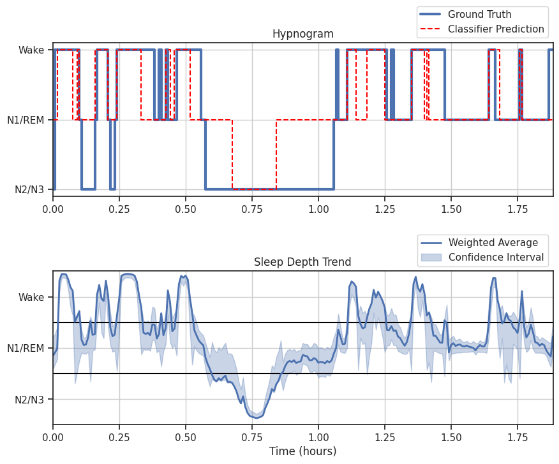* | *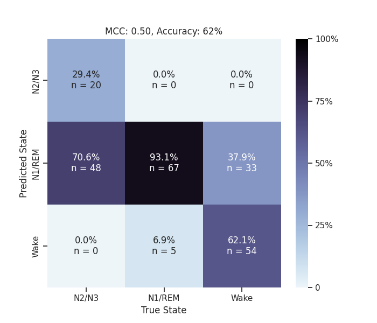* |
| *5* | *1 wk* | *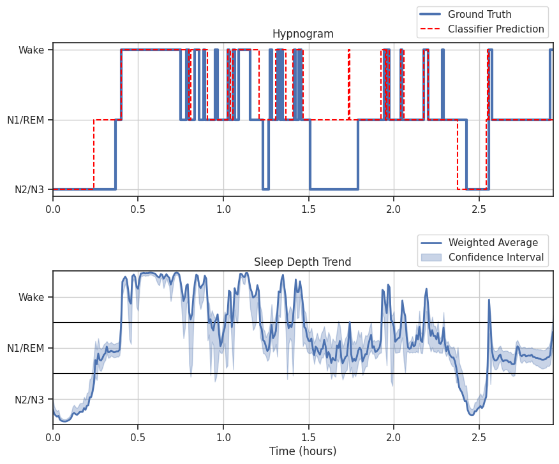* | *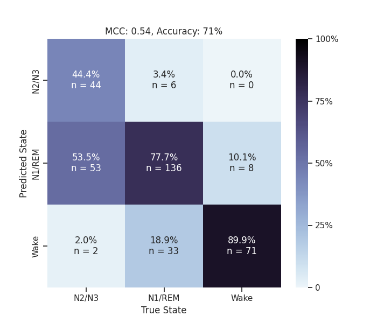* |
| *6* | *1 mos* | *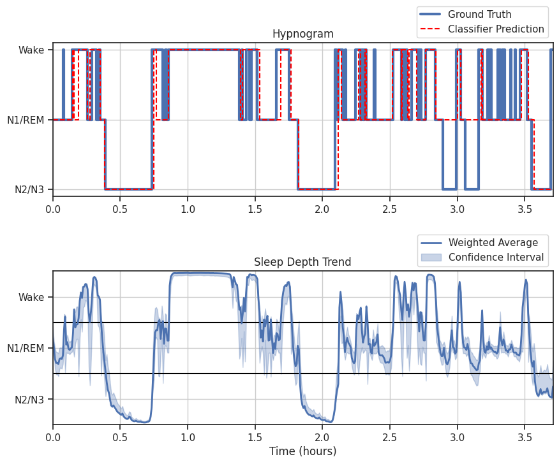* | *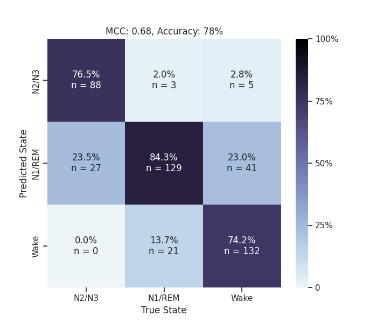* |
| *7* | *3 mos* |  |  |
| *8* | *1 mos* |  |  |
| *9* | *3 wks* |  |  |
| *10* | *1 mos* |  |  |
| *11* | *1 mos* |  |  |
| *12* | *5 mos* |  |  |
| *13* | *4 mos* |  |  |
| *14* | *6 mos* |  |  |
| *15* | *4 mos* |  |  |
| *16* | *1 mos* |  |  |
| *17* | *3 mos* |  |  |
| *18* | *3 mos* |  |  |
| *19* | *3 mos* |  |  |
| *20* | *9 mos* |  |  |
| *21* | *1 mos* |  |  |
| *22* | *3 mos* |  |  |
| *23* | *1 mos* |  |  |
| *24* | *3 mos* |  |  |
| *25* | *1 mos* |  |  |
| *26* | *3 mos* |  |  |
| *27* | *4 mos* |  |  |
| *28* | *12 mos* |  |  |
| *29* | *3 mos* |  |  |
| *30* | *1 mos* |  |  |
| *31* | *17 mos* |  |  |
| *32* | *18 mos* |  |  |
| *33* | *11 mos* |  |  |

**User Experience Questionnaire (S11)**

|  |  |
| --- | --- |
| 1. Did you encounter any issues while using the pants? | Yes / No |
| 1. If yes, describe. |  |
| 1. Would you use the pants again? | Yes / No |
| 1. If not, why? |  |
| 1. Is there any occasion you could feel discomfort using the pants? | Yes / No |
| 1. If yes, in which occasion and why? |  |
| 1. Are you confident the pants are safe in all circumstances? | Yes / No |
| 1. If not, describe your concern. |  |
| 1. Do you like the materials of the pants? | Yes / No |
| 1. If no, why? |  |
| 1. Did you notice any marks on the skin? | Yes / No |
| 1. If yes, describe how. |  |
| 1. Do you feel that the use of pants affected the infant’s behaviour? | Yes / No |
| 1. If yes, describe how. |  |
| 1. How do you observe the comfort of use from your infant’s perspective? |  |
| 1. Do you have any comments, concerns, or improvement suggestions related to the product? |  |

**Results of Training the Classifier with Subject-Wise Normalisation (S12)**

Opting for subject-wise z-score normalisation rather than global normalisation allows better control of the variance between sleep recordings; however, it comes at the cost of losing some classifier generalisability. For instance, subject-wise normalization requires that the given recording is long enough with sufficiently balanced sleep state distribution, which may not be the case with brief or otherwise disturbed sleep. By using the hybrid normalisation technique, replacing global z-score normalisation with subject-wise z-score normalisation, we conducted a similar assessment of the classifier performance as in the main paper.

The results (below) demonstrated improved classification accuracy compared to the classifier results in the main paper, where global z-score normalisation was used. The group-level results indicate that subject-wise z-score normalisation may enhance overall performance, improving both accuracy and MCC. Particularly, such classifiers perform better in distinguishing light sleep.

**Classifier Result CSV: Column Definitions and Explanations (S13)**

**Time**: This column represents the timestamp in POSIX time format, indicating the time at which the classification was performed.

**Logit prob 0**: This column contains the classifier output value for class 0. It represents the probability assigned by the classifier to the sample belonging to class 0.

**Logit prob 1**: This column contains the classifier output value for class 1. It represents the probability assigned by the classifier to the sample belonging to class 1.

**Logit prob 2:** This column contains the classifier output value for class 2. It represents the probability assigned by the classifier to the sample belonging to class 2.

Logit probs can be transformed into output probabilities with the Softmax operation.

Sleep Class : argmax_i (Logit prob i)

The sleep class is determined by selecting the index (i) with the highest logit probability, making use of the argmax operation. In simpler terms, with the Softmax operation identifies the sleep class with the highest likelihood based on the calculated logit probabilities.

**References**

1. Pedregosa F, Varoquaux G, Gramfort A, et al. Scikit-learn: Machine Learning in Python. June 2018. doi:10.48550/arXiv.1201.0490
